# HLA-II Expression Marks Activated and Clonally Expanded CD8^+^ T Cells and Improves Tumor-Reactivity Prediction Across Human Cancers

**DOI:** 10.3390/ijms27188338

**Published:** 2026-09-19

**Authors:** Xue Mi, Jinghua Zhu, Zhu Dai, Ruyue Yang, Dongmei Tian, Guochun Cao, Zhongdang Xiao

**Affiliations:** 1 State Key Laboratory of Digital Medical Engineering, School of Biological Science and Medical Engineering, Southeast University, Nanjing 210096, China; 230218235@seu.edu.cn (X.M.); 230239536@seu.edu.cn (Z.D.); 230249056@seu.edu.cn (R.Y.); 220255327@seu.edu.cn (D.T.); 2 Department of Medical Oncology, Jiangsu Cancer Hospital, Jiangsu Institute of Cancer Research, The Affiliated Cancer Hospital of Nanjing Medical University, Baiziting 42, Nanjing, 210009, China; zhujinghua1995@163.com

**Keywords:** biomedical engineering, deep learning, TCR-epitope, single-cell, HLA-II^+^ CD8^+^ T cell

## Abstract

Tumor-infiltrating CD8^+^ T cells are transcriptionally heterogeneous, and robust features linking their clonal dynamics to experimentally defined tumor reactivity remain incompletely characterized. We integrated single-cell RNA sequencing (scRNA-seq) and paired T-cell receptor sequencing (scTCR-seq) data from 31 single-cell immune datasets to characterize CD8^+^ T-cell states associated with clonal expansion and tumor reactivity. Across cancers, HLA-II-expressing CD8^+^ T cells were enriched in activated, cytotoxic, proliferative, exhausted, and terminally differentiated states and were preferentially associated with expanded TCR clonotypes. Pseudotime analysis indicated that HLA-II expression occurs along late differentiation states. HLA-DR blockade reduced CD25 upregulation in activated mixed PBMC and purified CD8^+^ T-cell cultures and attenuated CellTrace CFSE dilution in purified CD8^+^ T cells. An HLA-II-associated CD8^+^ T-cell signature showed cancer-type-dependent survival associations, including longer overall survival in several cohorts. To evaluate its contribution to tumor-reactivity prediction, an expansion-pretrained DNN was adapted using experimentally annotated tumor-reactive and non-tumor-reactive clonotypes from six pancreatic ductal adenocarcinoma samples and evaluated in 2225 functionally annotated CD8^+^ T cells from three held-out samples. Adding six HLA-II genes improved ROC-AUC across three feature panels from 0.540 to 0.781, 0.626 to 0.732, and 0.838 to 0.878, respectively, and improved PR-AUC from 0.570 to 0.739, 0.640 to 0.718, and 0.799 to 0.869. These findings identify HLA-II expression as a feature of activated and clonally expanded CD8^+^ T-cell states and support the complementary value of HLA-II-associated transcriptional features for prioritizing candidate tumor-reactive TCRs.

## 1. Introduction

The tumor microenvironment (TME) contains heterogeneous populations of tumor-infiltrating lymphocytes (TILs), among which tumor-reactive T cells are central mediators of cancer immune surveillance and therapeutic responses. Immune checkpoint inhibitors have achieved broad clinical application across numerous malignancies [1], although durable responses remain limited to a subset of patients. Adoptive cell therapy (ACT), particularly T-cell receptor-engineered T-cell (TCR-T) therapy [2], provides an alternative strategy for enhancing antitumor immunity through the transfer of antigen-specific T cells [3,4,5]. However, identifying tumor-reactive T-cell clonotypes generally requires peptide-stimulation assays, cytokine-release measurements, tumor-cell coculture, or ex vivo expansion [6]. These experimental procedures provide essential functional evidence but remain difficult to implement at the scale required for rapid therapeutic prioritization. The integration of single-cell RNA sequencing (scRNA-seq) with paired T-cell receptor sequencing (scTCR-seq) offers an opportunity to identify transcriptional and clonotypic features that can guide the selection of candidate tumor-reactive T cells for subsequent functional validation.

The T-cell receptor determines antigen specificity through recognition of peptide–major histocompatibility complex (pMHC) molecules and plays a central role in T-cell selection, activation, and differentiation [7,8,9]. Most human T cells express an αβ TCR heterodimer, with antigen-recognition diversity generated through V(D)J recombination and junctional nucleotide insertion and deletion [9,10,11,12]. The complementarity-determining region 3 (CDR3) of the TCRα and TCRβ chains constitutes the most variable region and makes a major contribution to antigen recognition [9]. Following antigen stimulation, T cells undergo transcriptional reprogramming, clonal expansion, and differentiation into cytotoxic effector, exhausted, or memory states [13,14,15]. Paired scRNA-seq and scTCR-seq profiling enables these cellular states to be linked directly to individual TCR clonotypes [16,17,18]. Clonal expansion provides evidence of antigen-experienced proliferation, whereas experimentally assigned tumor-reactivity labels provide functional evidence of recognition of autologous tumor cells. These two levels of evidence were therefore evaluated separately in the present study: large-scale clonotype data were first used to identify transcriptional features associated with expansion, followed by evaluation in TCR clonotypes with experimentally defined tumor-reactive or non-tumor-reactive annotations.

The integration of TCR sequences with gene expression data related to specific functional traits offers substantial synergistic effects, providing a comprehensive characterization of specific T-cell populations. Previous studies have shown that T cells responding to distinct antigens can exhibit divergent phenotypic and functional states, even when derived from the same pathogen [19]. Mapping transcriptomic expression to T-cell clonotypes facilitates the identification of functional subclusters undergoing clonal expansion, which provides evidence of antigen-driven proliferation [17] but does not establish tumor specificity. Expanded intratumoral clonotypes may recognize tumor antigens, viral antigens, microbial antigens, self-antigens, or other inflammatory stimuli. We therefore examined HLA-II expression first as a feature of activated and clonally expanded CD8^+^ T cells and subsequently evaluated its association with tumor reactivity in independently annotated datasets.

Several transcriptional markers and gene-expression signatures have been proposed to prioritize tumor-reactive T cells, including combinations involving CD39/ENTPD1, IL7R, CXCL13, CD40LG and GZMB [13,19,20,21,22]. Nevertheless, the phenotypic heterogeneity of tumor-infiltrating CD8^+^ T cells across patients and cancer types limits the generalizability of individual markers. HLA class II molecules are principally involved in antigen presentation to CD4^+^ T cells, but activated human CD8^+^ T cells can also express HLA-II genes, including members of the HLA-DR, HLA-DP, and HLA-DQ families [23]. The biological and predictive significance of this transcriptional program in tumor-infiltrating CD8^+^ T cells remains incompletely characterized. In this study, HLA-II refers collectively to the human HLA-DR, HLA-DP, and HLA-DQ gene families, whereas HLA-DR is used specifically for HLA-DR expression or HLA-DR-directed experimental intervention. The term MHC-II is reserved for pathway-level or intercellular signaling analyses.

Here, we integrated scRNA-seq and paired scTCR-seq data from 31 single-cell immune datasets to construct a pan-cancer T-cell atlas and systematically characterize HLA-II-associated CD8^+^ T-cell states. We examined the relationship of HLA-II expression with activation, cytotoxicity, differentiation state, tissue distribution, and TCR clonal expansion, and evaluated inferred MHC-II-associated communication between CD8^+^ and CD4^+^ T-cell populations. The functional contribution of HLA-DR was further investigated by blockade experiments in both activated mixed-PBMC cultures and purified CD8^+^ T-cell cultures. We also evaluated the clinical associations of an HLA-II-associated CD8^+^ T-cell signature across TCGA cancer cohorts. Finally, we developed an expansion-pretrained deep-learning framework and adapted it using experimentally annotated tumor-reactive and non-tumor-reactive TCR clonotypes from pancreatic ductal adenocarcinoma [24]. By comparing established transcriptional feature panels with and without six HLA-II genes in held-out patient samples, we assessed the complementary contribution of HLA-II-associated transcriptional features to tumor-reactivity prediction. Together, this framework connects large-scale analysis of activated and clonally expanded CD8^+^ T-cell states with functional experiments and experimentally annotated TCR data, providing a strategy for prioritizing candidate tumor-reactive TCRs for downstream validation.

## 2. Results and Discussion

### 2.1. Construction of a Pan-Cancer Single-Cell Atlas of T Cells

**Figure 1:**
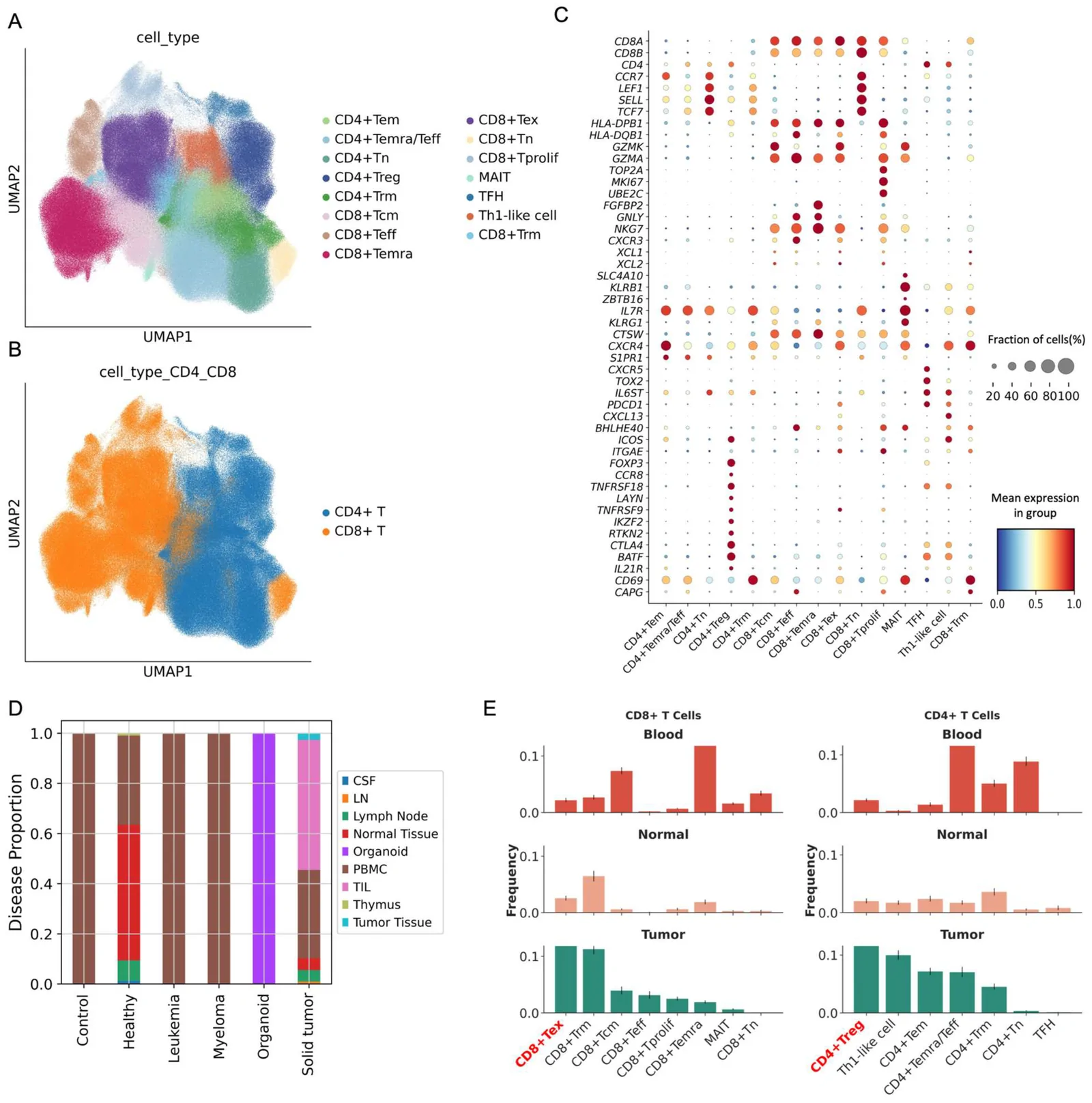
Construction and characterization of the pan-cancer single-cell T-cell atlas. The integrated atlas comprised 31 publicly available studies, 445 biological samples from 190 donors, 22 malignancies and healthy controls, nine anatomical sources and tissue compartments, and 858,417 quality-controlled T cells with paired transcriptomic and TCR profiles. (**A**) UMAP visualization of the integrated T-cell atlas, showing 15 major functional subsets within the CD4^+^ and CD8^+^ T-cell lineages. Transcriptomic profiles were integrated using scAtlasVAE [25], and paired transcriptomic and TCR information was represented using TCR-DeepInsight [26]. (**B**) UMAP visualization showing the overall distribution of CD4^+^ and CD8^+^ T cells within the integrated embedding space. (**C**) Expression of representative lineage and functional marker genes across the annotated T-cell subsets. Dot size represents the proportion of cells expressing each gene, and color intensity indicates the normalized average expression level. (**D**) Distribution of cells across disease states and anatomical sources, including tumor tissue, peripheral blood mononuclear cells, lymph nodes, cerebrospinal fluid, and other tissue compartments. (**E**) Relative frequencies of CD8^+^ T-cell subsets (left) and CD4^+^ T-cell subsets (right) across peripheral blood, adjacent normal tissue, and tumor tissue. Subset frequencies were calculated independently within each biological sample. Bars represent the mean, and error bars indicate the standard error of the mean across independent biological samples.

To characterize the transcriptional and clonal diversity of T cells across cancers, we integrated 31 publicly available studies containing paired single-cell RNA sequencing (scRNA-seq) and single-cell TCR sequencing (scTCR-seq) data (Supplementary Figure S1A). The resulting atlas encompassed 22 malignancies and healthy controls across nine anatomical sources and tissue compartments, including tumor tissues, peripheral blood mononuclear cells, lymph nodes, and cerebrospinal fluid (Figure 1D). In total, the atlas included 445 biological samples from 190 donors. In addition to the CD8^+^ T-cell landscape used for the principal downstream analyses, a corresponding CD4^+^ T-cell landscape was constructed to characterize CD4^+^ T-cell heterogeneity and provide a reference for the subsequent analysis of communication between CD8^+^ and CD4^+^ T-cell populations (Supplementary Figure S1B). After quality control, the atlas comprised 858,417 T cells with paired transcriptomic profiles and full-length paired TCRα and TCRβ sequences.

To reduce technical variation among studies while preserving biological heterogeneity, the transcriptomic data were integrated using scAtlasVAE [25], and the paired TCR and transcriptomic information was represented using TCR-DeepInsight [26]. Using the top 5000 highly variable genes for dimensionality reduction, we resolved 15 major T-cell subsets, which were annotated according to established lineage and functional markers (Figure 1A–C). CD8^+^ T cells were classified into naïve T cells (CD8^+^ Tn), central memory T cells (CD8^+^ Tcm), effector T cells (CD8^+^ Teff), terminally differentiated effector memory T cells (CD8^+^ Temra), resident memory T cells (CD8^+^ Trm), proliferating T cells (CD8^+^ Tprolif), exhausted T cells (CD8^+^ Tex), and mucosal-associated invariant T cells (MAIT). The CD4^+^ T-cell compartment included naïve T cells (CD4^+^ Tn), effector memory T cells (CD4^+^ Tem), terminally differentiated effector memory/effector T cells (CD4^+^ Temra/Teff), regulatory T cells (CD4^+^ Treg), resident memory T cells (CD4^+^ Trm), follicular helper T cells (Tfh), and Th1-like cells.

The relative abundance of these T-cell states varied across tissue compartments (Figure 1E). CD8^+^ exhausted T cells accounted for approximately 12% of the CD8^+^ T-cell compartment in tumor samples on average but were less abundant in peripheral blood and adjacent normal tissues. In contrast, CD8^+^ terminally differentiated effector memory T cells were more prevalent in peripheral blood. Tumor samples also showed higher mean proportions of CD4^+^ regulatory and Th1-like cells than peripheral blood and adjacent normal tissues. Together, these data establish a pan-cancer single-cell T-cell atlas for subsequent analyses of HLA-II expression, T-cell activation, and clonal expansion.

### 2.2. Pan-Cancer Analysis of Tumor-Associated T-Cell Activation, HLA-II Expression, and CD8^+^ T-Cell Clonal Expansion

**Figure 2:**
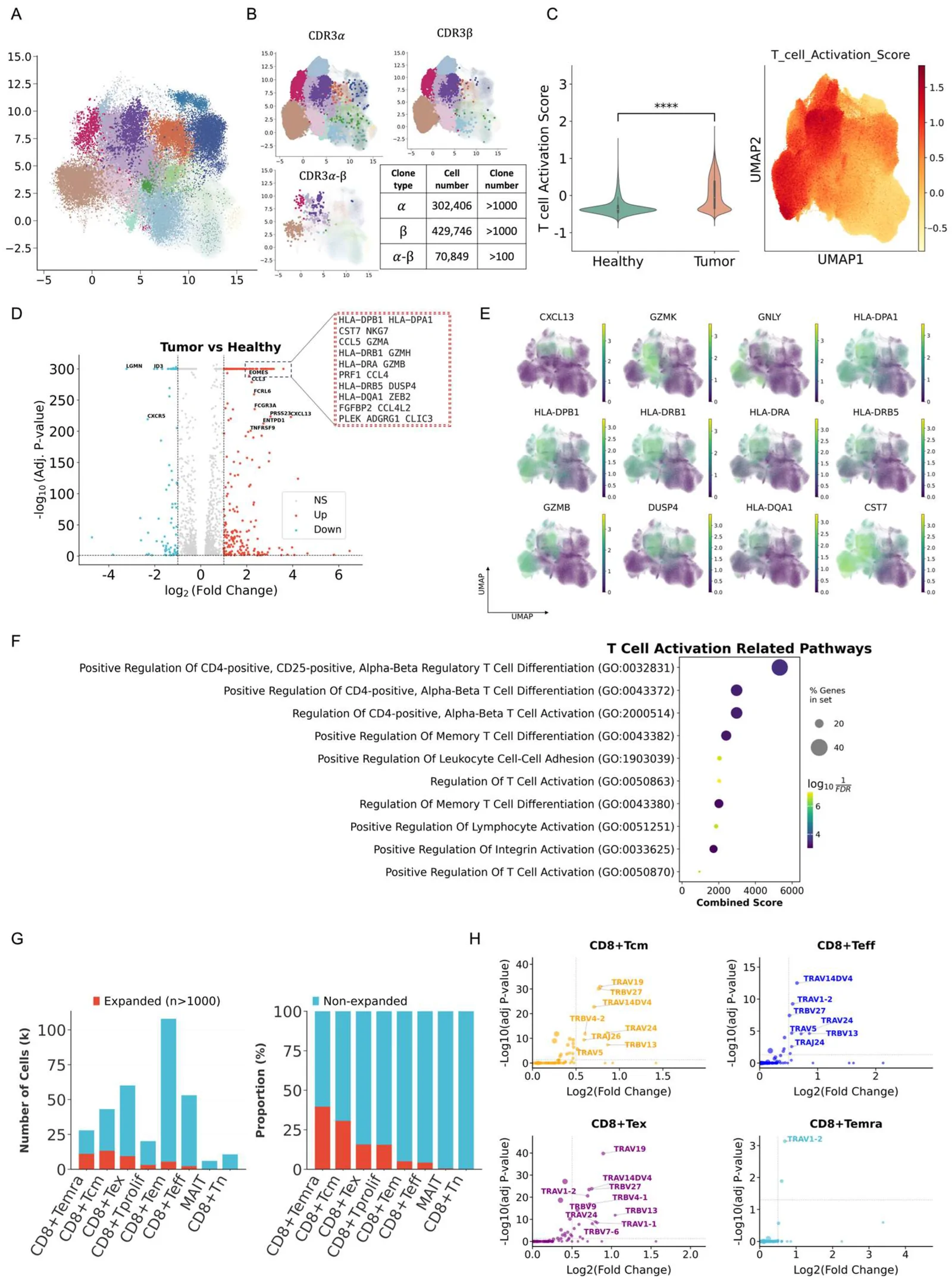
Tumor-Associated T-Cell Activation, HLA-II Expression, CD8^+^ T-Cell Clonal Expansion, and TCR Repertoire Features. The analyses were performed using the integrated pan-cancer T-cell atlas. The tumor-derived subset comprised 603,708 quality-controlled T cells from 335 biological samples and 139 donors; panel-specific cell and clonotype counts are indicated where applicable. (**A**) Clonotype-level UMAP representation generated by aggregating the transcriptomic embedding coordinates of cells sharing the same paired TCR clonotype. Each point represents one clonotype. Each point represents one clonotype. Different colors indicate distinct clusters, see Figure 1A for reference. (**B**) Distribution and quality-control summary of recovered CDR3α, CDR3β, and paired CDR3αβ sequences. The accompanying table reports the corresponding cell and clonotype counts. (**C**) Comparison of cell-level T-cell activation scores between healthy-control and tumor-derived samples. Each observation represents one cell; **** indicates *p* < 0.0001 using a two-sided Wilcoxon rank-sum test. Activation scores are also projected onto the single-cell transcriptomic UMAP. (**D**) Volcano plot showing differentially expressed genes between clonotype-aggregated expression profiles derived from tumor and healthy-control samples. Each expression profile represents one sample-specific clonotype. Red and blue points indicate significantly upregulated and downregulated genes, respectively, using thresholds of |$log_{2}\;fold\;change$| > 1 and adjusted *p* < 0.05. (**E**) UMAP projections showing the expression of representative activation-, cytotoxicity-, and HLA-II-associated genes, including CXCL13, GZMK, GNLY, GZMB, DUSP4, CST7, HLA-DPA1, HLA-DPB1, HLA-DQA1, HLA-DRA, HLA-DRB1, and HLA-DRB5. (**F**) Gene Ontology enrichment analysis of genes upregulated in tumor-derived clonotype-aggregated profiles. The horizontal axis represents the combined enrichment score, dot size indicates the percentage of genes represented in each gene set, and color denotes statistical significance based on the false discovery rate. (**G**) Distribution of cells belonging to highly expanded and non-expanded clonotypes across CD8^+^ T-cell subsets. Highly expanded clonotypes were defined within each biological sample as clonotypes represented by more than 1000 cells, whereas clonotypes represented by 1000 cells or fewer were classified as non-expanded. The left panel shows absolute cell counts, and the right panel shows the corresponding proportions within each CD8^+^ T-cell subset. (**H**) One-vs-rest analysis of TCR gene-segment usage at the unique-clonotype level. For each target population—CD8^+^ Tcm, Teff, Tex, or Temra—the usage frequency of each TRAV, TRAJ, TRBV, and TRBJ segment among clonotypes assigned to that population was compared with its frequency among clonotypes assigned to all remaining T-cell subsets. Clonotypes were assigned to a single transcriptional population according to the majority annotation of their constituent cells. The horizontal axis represents the log_2_ fold change in gene-segment usage frequency between the target population and the remaining T-cell populations, and the vertical axis represents −log_10_ of the adjusted *p* value. Significantly enriched gene segments are labeled. Enrichment was evaluated using two-sided Fisher’s exact tests, with *p* values adjusted for multiple testing using the Benjamini–Hochberg method.

To characterize the global distribution of T-cell clonotypes, we mapped TCR-defined clonotypes, identified on the basis of paired CDR3α and CDR3β sequences and the corresponding V/J gene usage, onto the transcriptomic embedding space. By aggregating the UMAP coordinates of cells belonging to the same clonotype, we generated a clonotype-level landscape of the T-cell populations (Figure 2A). Clonotype phenotypes were assigned using a majority-voting strategy based on the dominant transcriptional annotation among their constituent cells. Quality control was subsequently applied to retain cells with recovered TCRα, TCRβ, or paired TCRαβ sequences (Figure 2B). We next compared T-cell activation states between tumor-derived and healthy-control samples. At the cell level, T-cell activation scores were higher in tumor-derived samples than in healthy-control samples (two-sided Wilcoxon rank-sum test, *p* < 0.0001; Figure 2C), with higher scores localized to specific regions of the transcriptomic embedding.

To identify tumor-associated transcriptional features, we performed differential expression analysis between clonotype-aggregated expression profiles derived from tumor and healthy-control samples (Figure 2D). Multiple effector- and cytotoxicity-associated genes, including GNLY, GZMK, GZMB, NKG7, and PRF1, were upregulated together with six HLA-II genes: HLA-DPA1, HLA-DPB1, HLA-DQA1, HLA-DRA, HLA-DRB1, and HLA-DRB5. Projection onto the CD8^+^ T-cell embedding showed that these HLA-II genes were concentrated within transcriptional regions that also exhibited activation- and cytotoxicity-associated features (Figure 2E). This concordant regional distribution was consistent with an association between HLA-II expression and activated, effector-related transcriptional states (Figure 2D,E). Pathway enrichment analysis further identified pathways related to T-cell activation, regulatory and memory T-cell differentiation, lymphocyte activation, and leukocyte cell–cell adhesion (Figure 2F).

To further examine clonal expansion patterns, we quantified the distribution of paired CDR3αβ clonotypes across CD8^+^ T-cell subsets. Clonotypes represented by more than 1000 cells within an individual biological sample were classified as highly expanded. The proportion of cells belonging to highly expanded clonotypes was greatest in the CD8^+^ Temra and CD8^+^ Tcm populations, with smaller proportions observed in the CD8^+^ Tex and CD8^+^ Tprolif populations (Figure 2G). One-vs-rest analysis of TCR gene-segment usage, performed at the sample-specific clonotype level, identified gene segments preferentially used by clonotypes assigned to the CD8^+^ Tcm, Teff, Tex, and Temra populations relative to clonotypes assigned to all remaining T-cell subsets (Figure 2H). Together, these analyses delineate tumor-associated activation and HLA-II-associated transcriptional features, alongside patterns of clonal expansion and T-cell-subset-associated TCR gene-segment usage.

### 2.3. HLA-II Expression Along the CD8^+^ T-Cell Differentiation-State Trajectory

**Figure 3:**
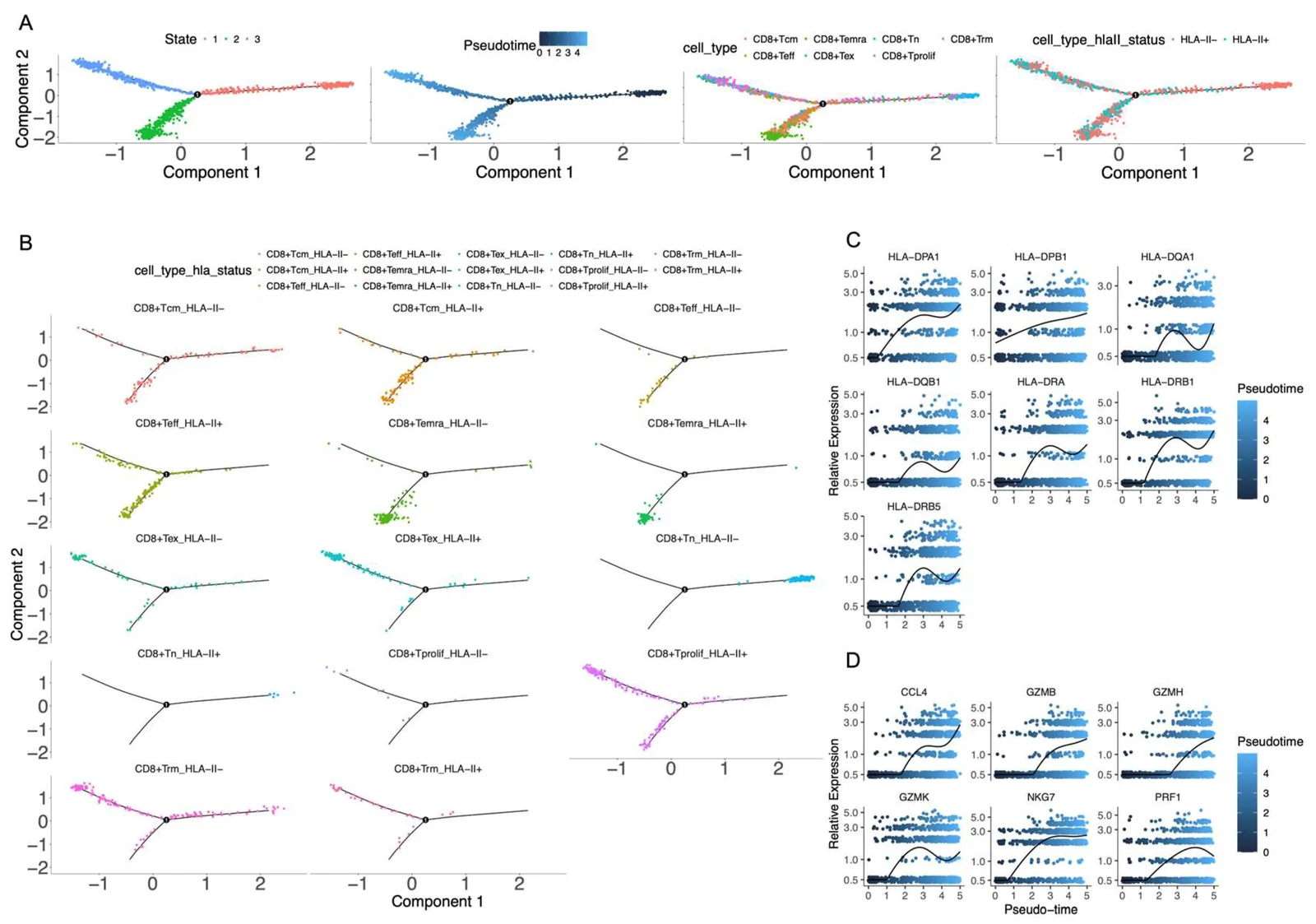
HLA-II Expression along the CD8^+^ T-Cell Differentiation-State Trajectory. (**A**) Differentiation-state trajectory of CD8^+^ T cells inferred using Monocle2. The subpanels, from top to bottom, show cells colored by trajectory state (States 1–3), pseudotime, T-cell subset, and HLA-II expression status (HLA-II^+^ versus HLA-II^−^). The inferred trajectory extends from a naïve-like region toward two branched states. The circled number ① indicates the trajectory branch point where cells differentiate into three separate states. (**B**) Distribution of individual CD8^+^ T-cell subsets and their corresponding HLA-II expression status along the trajectory. HLA-II^+^ cells are preferentially distributed at intermediate and later pseudotime positions. (**C**) Smoothed expression trends of HLA-DPA1, HLA-DPB1, HLA-DQA1, HLA-DQB1, HLA-DRA, HLA-DRB1, and HLA-DRB5 over pseudotime. Points represent normalized expression values of individual cells, and solid lines represent expression trends fitted using generalized linear models. (**D**) Smoothed pseudotime-associated expression trends of the cytotoxicity- and effector-associated genes CCL4, GZMB, GZMH, GZMK, NKG7, and PRF1.

To characterize the differentiation-state organization of CD8^+^ T cells within the tumor microenvironment, we performed pseudotime trajectory analysis. The inferred trajectory extended from an early naïve-like state (State 1) toward two branched states (States 2 and 3) (Figure 3A). Projection of CD8^+^ T-cell subsets onto the trajectory showed that CD8^+^ Tn cells were primarily localized near the root, whereas CD8^+^ Tex, Temra, and Tprolif populations were predominantly distributed at later pseudotime positions (Figure 3A). When HLA-II expression status was mapped onto the trajectory, HLA-II^−^ CD8^+^ T cells were more frequently located near the early region, whereas HLA-II^+^ CD8^+^ T cells were enriched at intermediate and later pseudotime positions, particularly along branches occupied by terminal effector and exhausted populations (Figure 3B). These results associate HLA-II expression with intermediate-to-late differentiation states of CD8^+^ T cells.

We next examined HLA-II gene-expression patterns over pseudotime. HLA-DPA1, HLA-DPB1, HLA-DQA1, HLA-DQB1, HLA-DRA, HLA-DRB1, and HLA-DRB5 showed higher expression at intermediate and later pseudotime positions (Figure 3C). Cytotoxicity- and effector-associated genes, including CCL4, GZMB, GZMH, GZMK, NKG7, and PRF1, also showed increased expression toward later positions along the trajectory (Figure 3D). Together, these patterns support an association between HLA-II expression and differentiated CD8^+^ T-cell states exhibiting cytotoxic and effector-related transcriptional features.

### 2.4. Predicted MHC-II Communication Is Increased in Tumor-Infiltrating T-Cell Populations

**Figure 4:**
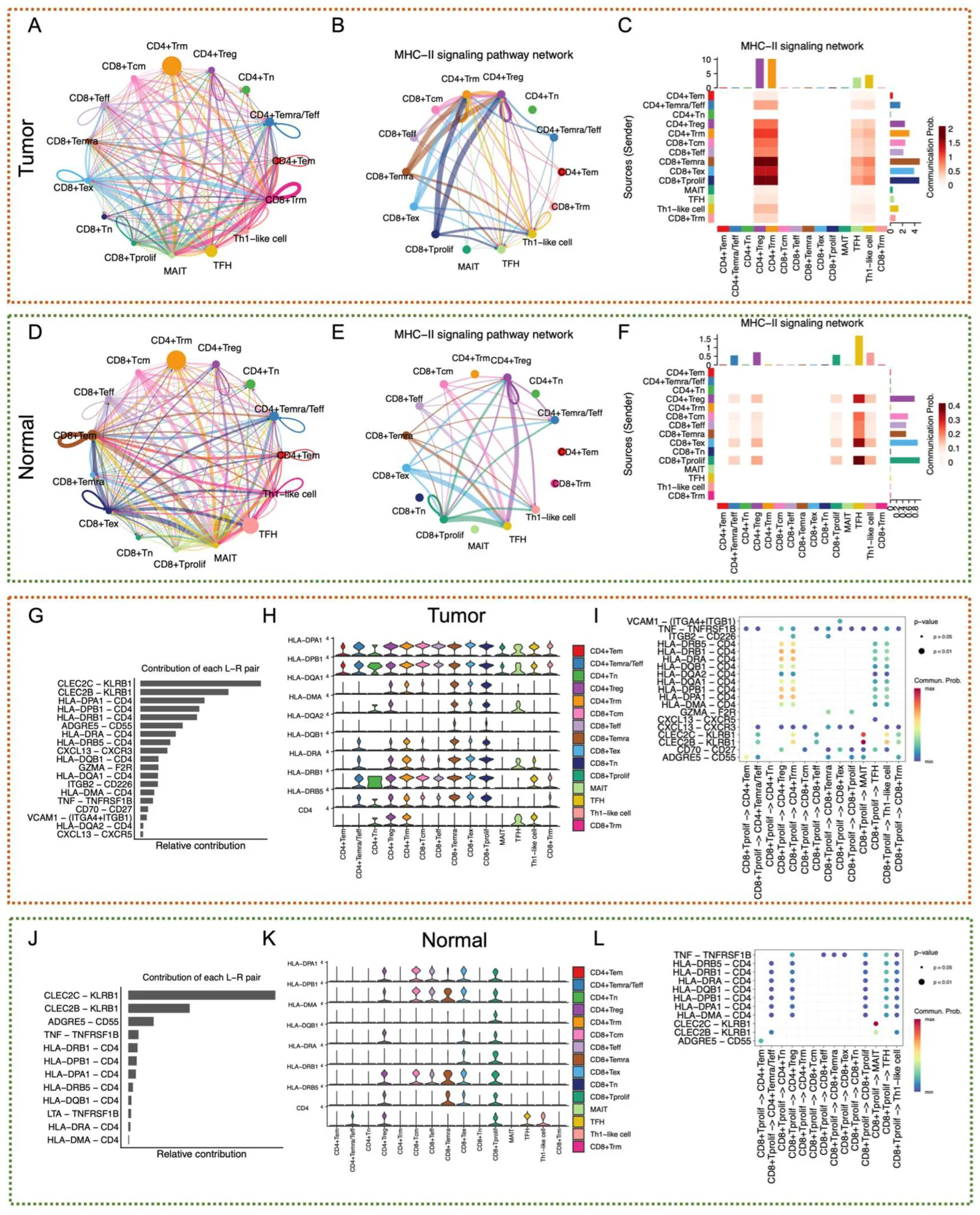
Tumor-Associated Differences in Predicted MHC-II Communication among T-Cell Populations. (**A**,**B**) Global communication network diagram among T-cell subpopulations (**A**) and the specific MHC-II signaling pathway network (**B**) within tumor tissues. Circles represent cell subpopulations, the thickness of the connecting lines indicates interaction strength, and arrows point toward the signal receivers (Targets). (**C**) Heatmap of communication probabilities for senders (Sources, vertical axis) and receivers (Targets, horizontal axis) within the MHC-II pathway in tumor tissues. (**D**,**E**) Global communication network diagram (**D**) and MHC-II signaling network (**E**) in corresponding normal tissues, illustrating a markedly contracted communication landscape compared to tumor tissues. (**F**) Heatmap of communication probabilities for the MHC-II pathway in normal tissues. (**G**,**J**) Bar charts comparing the relative contributions of individual ligand–receptor pairs (L-R pairs) to the MHC-II signaling pathway in tumor (**G**) and normal (**J**) tissues. (**H**,**K**) Violin plots displaying the expression distribution of key ligand genes involved in the MHC-II pathway, such as HLA-DRA and HLA-DPA1, and their receptor CD4 across various subpopulations in tumor (**H**) and normal (**K**) tissues. (**I**,**L**) Dot plots quantitatively comparing the communication probabilities of specific ligand–receptor pairs across different cell pairs in tumor (**I**) and normal (**L**) tissues. The size of the dots represents the statistical significance (*p* value), and the color intensity denotes the communication probability.

CellChat analysis was performed using 603,708 tumor-derived T cells from 335 biological samples and 139 donors and 254,709 normal-tissue-derived T cells from 110 biological samples and 51 donors. To reduce the influence of differences in cell numbers and cellular composition, direct comparisons were restricted to T-cell subsets represented in both conditions. Within each shared subset, cells from the condition containing the larger population were downsampled to match the cell number in the smaller population before communication inference. Analysis of the resulting cell-number-balanced datasets showed that the predicted number and aggregate strength of interactions were generally greater in tumor tissues than in normal tissues. Normal-tissue networks showed fewer and more evenly distributed interactions (Figure 4D,E), whereas tumor tissues exhibited broader and more directional predicted communication among T-cell subsets (Figure 4A,B). These differences were particularly evident in the MHC-II signaling pathway. Predicted MHC-II communication probabilities were generally low across T-cell populations in normal tissues (Figure 4F) but higher in tumor tissues, where CD8^+^ T-cell subsets were identified as prominent predicted senders toward multiple CD4^+^ T-cell populations (Figure 4C). CD8^+^ Tex and CD8^+^ T_prolif_ showed the strongest predicted outgoing activity within this pathway.

We next examined the expression of representative HLA-II ligand genes across T-cell subsets. HLA-DRA, HLA-DPA1, HLA-DPB1, and HLA-DRB1 showed low expression in CD8^+^ T-cell populations from normal tissues but higher expression in tumor-derived CD8^+^ T cells, particularly in the CD8^+^ Tex and CD8^+^ Tprolif populations (Figure 4H,K). These expression patterns were consistent with the differences in predicted MHC-II communication. In tumor tissues, HLA-DPA1–CD4, HLA-DPB1–CD4, HLA-DRA–CD4, and HLA-DRB1–CD4 made substantial contributions to the inferred communication network (Figure 4G). By comparison, alternative ligand–receptor interactions, including CLEC2C–KLRB1 and ADGRE5–CD55, were more prominent in the normal-tissue network (Figure 4J).

Predicted interaction probabilities for most HLA-II–CD4 ligand–receptor pairs were low or not statistically significant in normal tissues (Figure 4L and Supplementary Figure S2), whereas multiple HLA-II–CD4 interactions were detected in tumor tissues (Figure 4I; permutation *p* < 0.05). The strongest predicted interactions originated from CD8^+^ Tex and CD8^+^ T_prolif_ populations and were directed toward CD4^+^ T-cell subsets. Together, the analysis of cell-number-balanced datasets identified a tumor-associated increase in predicted MHC-II pathway communication involving HLA-II-expressing CD8^+^ T-cell populations.

### 2.5. HLA-DR Blockade Attenuates CD25 Upregulation and Proliferation in Activated CD8^+^ T Cells

To examine the effects of HLA-DR blockade on CD8^+^ T-cell activation, parallel experiments were performed using mixed PBMC cultures and CD8^+^ T cells purified from PBMCs by negative selection. Flow-cytometric assessment confirmed successful CD8^+^ T-cell enrichment, with the percentage of CD8^+^ cells increasing from 49.2% before isolation to 97.0% after negative selection (Supplementary Figure S3). Cells were maintained without stimulation, stimulated with anti-CD3/CD28 beads and IL-2, or stimulated in the presence of the HLA-DR-blocking antibody L243.

**Figure 5:**
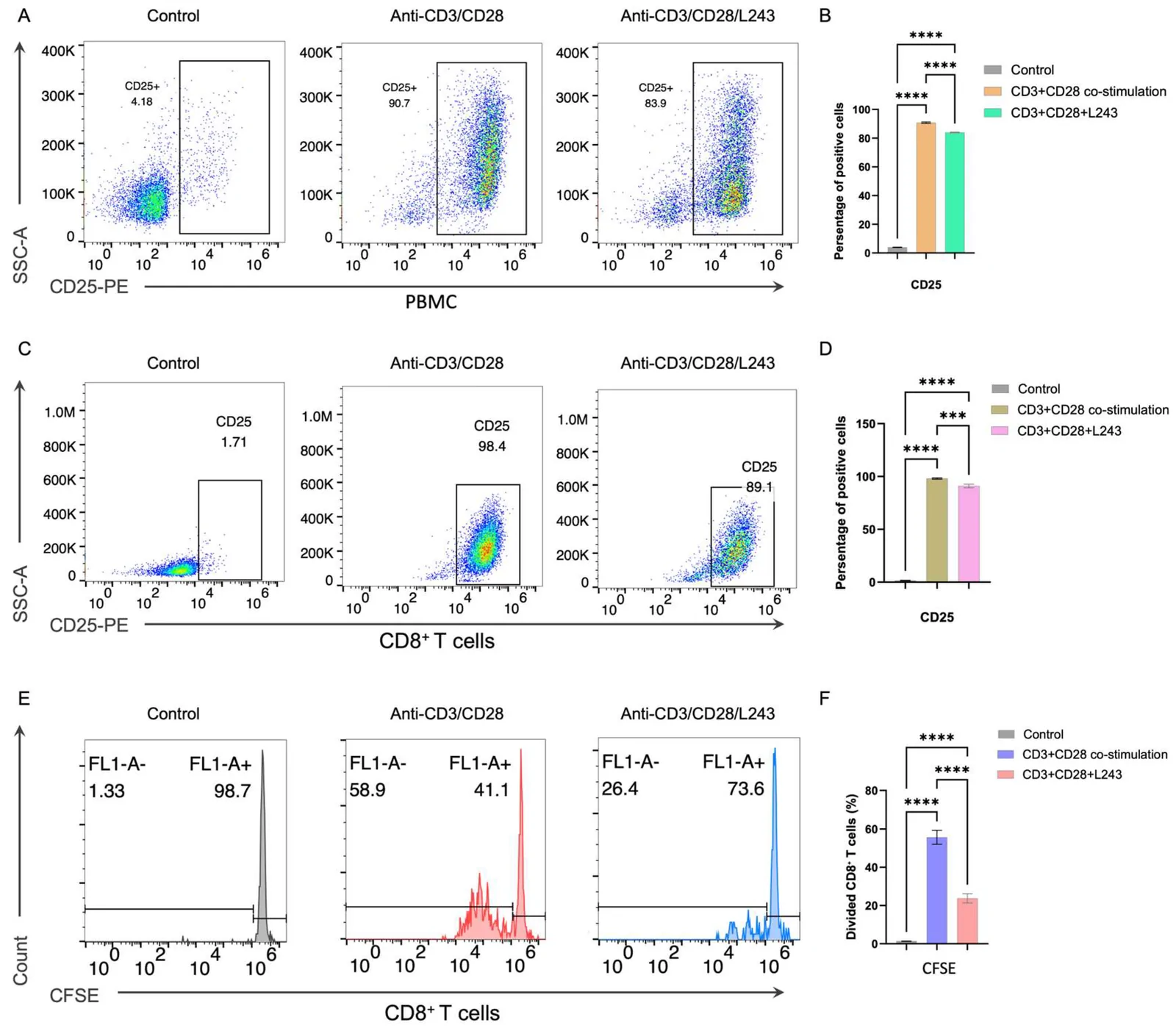
Effects of HLA-DR blockade on CD25 expression and proliferation in mixed PBMC and purified CD8^+^ T-cell cultures. (**A**) Representative flow-cytometry plots showing CD25 expression within gated CD8^+^ singlet lymphocytes from mixed PBMC cultures under the unstimulated control, anti-CD3/CD28 plus IL-2 stimulation, and anti-CD3/CD28 plus IL-2 stimulation with the HLA-DR-blocking antibody L243 conditions. Values shown beside the gates indicate the percentage of CD25^+^ cells within the gated CD8^+^ singlet lymphocyte population. Color intensity represents event density, with warmer colors indicating higher cell density. (**B**) Statistical bar graph showing the percentage of CD25^+^ cells in mixed PBMC cultures based on three independent experiments. Bars and error bars represent the mean ± standard error of the mean (SEM). Statistical significance was evaluated using one-way analysis of variance (ANOVA) followed by Tukey’s multiple-comparison test (**** indicates *p* < 0.0001). (**C**) Representative flow-cytometry plots showing CD25 expression in purified CD8^+^ T-cell cultures under the same three treatment conditions. Values shown beside the gates indicate the percentage of CD25^+^ cells within the gated CD8^+^ singlet population. (**D**) Statistical bar graph showing the percentage of CD25^+^ cells in purified CD8^+^ T-cell cultures based on three independent experiments. Bars and error bars represent the mean ± standard error of the mean (SEM). Statistical significance was evaluated using one-way analysis of variance (ANOVA) followed by Tukey’s multiple-comparison test (**** indicates *p* < 0.0001, *** indicates *p* < 0.001). (**E**) Representative CellTrace CFSE dilution profiles of purified CD8^+^ T cells under the three treatment conditions. (**F**) Statistical bar graph showing the percentage of proliferating CFSE-low CD8^+^ T cells based on three independent experiments. Bars and error bars represent the mean ± standard error of the mean (SEM). Statistical significance was evaluated using one-way analysis of variance (ANOVA) followed by Tukey’s multiple-comparison test (**** indicates *p* < 0.0001). The complete flow-cytometry gating hierarchy is shown in Supplementary Figure S4.

In mixed PBMC cultures, CD3/CD28 stimulation markedly increased the percentage of CD25^+^ cells within the gated CD8^+^ singlet lymphocyte population, whereas the addition of L243 reduced CD25 upregulation (Figure 5A,B). A consistent reduction in CD25 expression was observed when the experiment was repeated using purified CD8^+^ T cells (Figure 5C,D). Thus, the reduction in CD25 expression following HLA-DR blockade was reproduced in the highly enriched CD8^+^ T-cell preparation, indicating that this effect was not solely dependent on non-CD8^+^ accessory cells present in mixed PBMC cultures.

We next examined CD8^+^ T-cell proliferation using CellTrace CFSE dilution. CD3/CD28 plus IL-2 stimulation induced extensive CFSE dilution in purified CD8^+^ T cells, whereas the addition of L243 reduced the proportion of divided cells relative to the stimulated group (Figure 5E,F). CD69 expression was evaluated separately in mixed PBMC and purified CD8^+^ T-cell cultures. In mixed PBMC cultures, the percentage of CD69^+^ cells within the gated CD8^+^ singlet lymphocyte population increased from 15.7% in the unstimulated group to 71.1% following CD3/CD28 plus IL-2 stimulation and remained high in the presence of L243 (82.0%; Supplementary Figure S5A,B). A similar pattern was observed in purified CD8^+^ T-cell cultures, in which the percentage of CD69^+^ cells increased from 4.72% to 88.4% following stimulation and reached 92.6% in the presence of L243 (Supplementary Figure S5C,D). Together, these results show that HLA-DR blockade attenuated CD25 upregulation and proliferative responses while CD69 expression remained elevated. The concordant CD25 results obtained in mixed PBMC and purified CD8^+^ T-cell cultures support a CD8^+^ T-cell-associated contribution of HLA-DR under these activation conditions.

### 2.6. Characteristics of the TCR Repertoire in CD8^+^ T Cells

**Figure 6:**
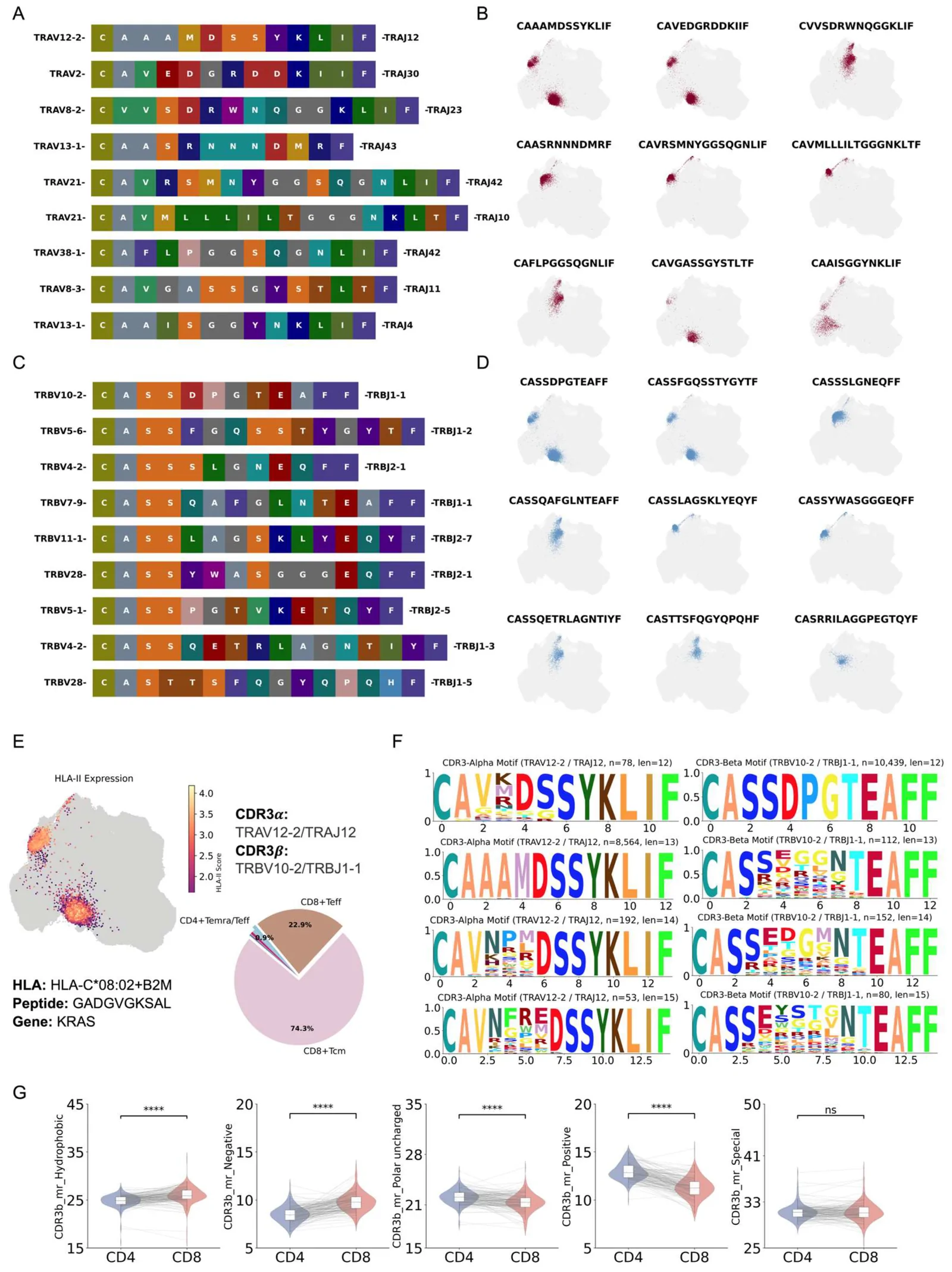
TCR Repertoire Characteristics and Physicochemical Sequence Analysis of HLA-II^+^ CD8^+^ T Cells. (**A**,**C**) Frequency distribution plots illustrating high-frequency gene rearrangement combinations in HLA-II^+^ CD8^+^ T cells, specifically for (**A**) TRAV-TRAJ and (**C**) TRBV-TRBJ. The bar lengths represent cell counts for specific V-J pairings, and colors correspond to distinct CDR3 amino acid sequence motifs. (**B**,**D**) UMAP projections displaying the distribution of dominant (**B**) CDR3α and (**D**) CDR3β clonotypes within the transcriptomic manifold, highlighting their spatial consistency with highly activated subpopulations. (**E**) Characterization of experimentally annotated KRAS G12D-reactive TCR clonotypes. The left UMAP plot shows the colocalization of HLA-II expression levels with specific TCR sequences, while the right pie chart quantifies the proportional distribution of clonotypes recognizing the KRAS G12D-derived peptide GADGVGKSAL presented by HLA-C*08:02 across subpopulations such as CD8^+^ Tcm and CD8^+^ Teff. (**F**) Sequence logos illustrating the conserved motifs of CDR3α and CDR3β sequences within dominant V-J combinations. Information entropy (measured in bits) represents the degree of amino acid conservation at specific positions. (**G**) Statistical comparison of the physicochemical properties of CDR3β sequences between CD8^+^ T cells (red) and CD4^+^ T cells (blue). Analyzed metrics include the hydrophobicity index, proportion of negatively charged residues, proportion of polar uncharged residues, and proportion of positively charged residues. Statistical significance was determined using the Wilcoxon rank-sum test (**** indicates *p* < 0.0001; ns indicates no significant difference).

To characterize TCR repertoire features associated with HLA-II^+^ CD8^+^ T cells, we analyzed V(D)J gene usage patterns across paired αβ TCR sequences. In the α chain, TRAV12-2/TRAJ12 and TRAV2/TRAJ30 were among the most frequently detected rearrangements (Figure 6A). In the β chain, TRBV10-2/TRBJ1-1 showed the highest usage frequency (Figure 6C). These dominant clonotypes exhibited clustered distributions within the UMAP space (Figure 6B,D) and overlapped with transcriptionally activated T-cell states, indicating an association between clonotype abundance and T-cell activation. We next examined CDR3 sequence conservation among highly represented clonotypes. Motif analysis of CDR3 sequences showed that both α- and β-chain clonotypes exhibited conserved amino acid residues at the flanking regions, whereas the central regions displayed substantially higher sequence variability, particularly among clonotypes carrying TRAV12-2 and TRBV10-2 rearrangements (Figure 6F). These conserved sequence features suggest preferential expansion of specific TCR clonotypes within tumor-associated CD8^+^ T-cell populations. To examine HLA-II expression in a defined tumor-antigen-specific setting, we analyzed experimentally annotated TCR clonotypes recognizing the KRAS G12D-derived peptide GADGVGKSAL presented by HLA-C*08:02. These KRAS G12D-reactive clonotypes were primarily distributed within the CD8^+^ T_cm_ and CD8^+^ T_eff_ populations and overlapped with regions characterized by HLA-II expression (Figure 6E). Within this antigen-annotated subset, the observed HLA-II expression was consistent with an association between HLA-II-associated transcriptional states and tumor-antigen-specific CD8^+^ T cells.

To characterize lineage-associated differences in TCR sequence properties, we compared the physicochemical characteristics of CDR3β sequences between CD8^+^ and CD4^+^ T cells. The results showed that the amino acid residues in the CDR3β region of CD8^+^ T cells exhibit a significantly higher hydrophobicity index and a greater proportion of negatively charged residues (*p* < 0.0001, Figure 6G). Conversely, the proportion of positively charged residues was significantly lower than in CD4^+^ T cells, a specific physicochemical preference pattern that was also observed in the CDR3α region (Supplementary Figure S6). These differences indicate distinct physicochemical characteristics of the CDR3 repertoires of CD8^+^ and CD4^+^ T cells. Furthermore, all subpopulations exhibited high conservation and distributional consistency in the total length of CDR3αβ and the length of the “mr” regions (Supplementary Figure S7). The stability of these length distributions indicates that despite significant differences in V(D)J gene preferences and physicochemical properties, the receptor repertoires maintain similar structural frameworks. In summary, HLA-II^+^ CD8^+^ T cells were associated with activated transcriptional states and exhibited distinct patterns of V–J gene usage, CDR3 sequence composition, and physicochemical properties. Analysis of the annotated KRAS G12D-reactive clonotypes further linked HLA-II expression to a defined tumor-antigen-specific T-cell population.

### 2.7. Pan-Cancer Prognostic Associations of the HLA-II-Associated CD8^+^ T-Cell Signature

**Figure 7:**
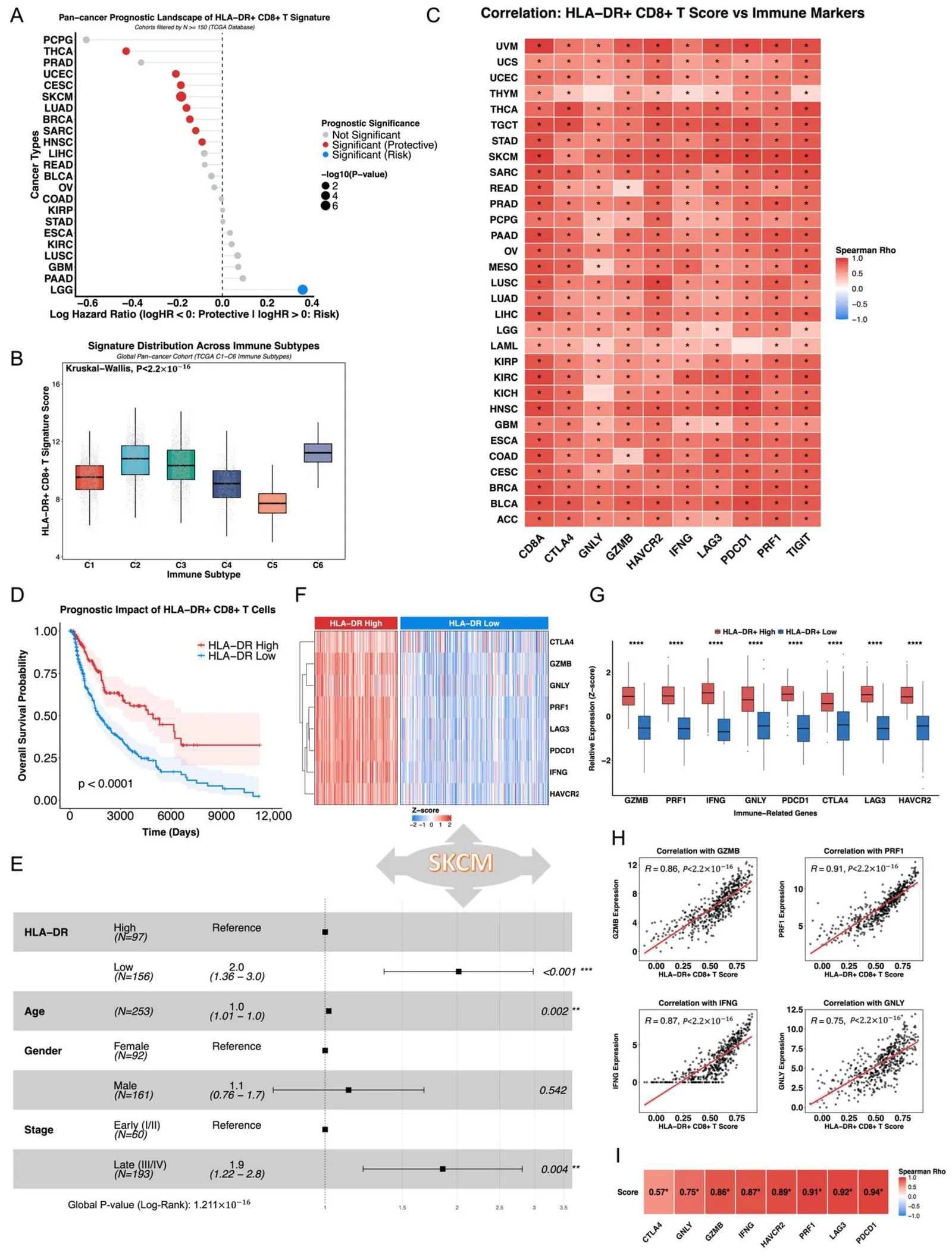
Pan-Cancer Survival and Immune Associations of the HLA-II-Associated CD8^+^ T-Cell Signature. (**A**) Forest plot illustrating the prognostic significance of the HLA-DR^+^ CD8^+^ T signature score across 23 cancer types from TCGA. The size of the points represents statistical significance (−${log}_{10}$ *p* value), while the position indicates the Log Hazard Ratio. Red points denote cancer types where the signature serves as a significant protective factor, such as SKCM, LUAD, and BRCA. (**B**) Box plot displaying the distribution of the signature score across six pan-cancer immune subtypes (C1–C6), with statistical significance determined by the Kruskal–Wallis test (*p* < ${2.2\times 10}^{-16}$). (**C**) Correlation heatmap showing the strength of positive associations (Spearman’s rho) between the signature score and cytotoxic markers such as GZMB and PRF1, as well as immune checkpoint genes including PDCD1 and LAG3 at a pan-cancer level (* indicates *p* < 0.05). (**D**–**I**) In-depth analysis of the skin cutaneous melanoma (SKCM) cohort. (**D**) Kaplan–Meier overall survival (OS) curves comparing high-score and low-score groups. (**E**) Multivariable Cox regression analysis of overall survival in the SKCM cohort, adjusted for age, sex, and clinical stage. The high-score group was used as the reference; the hazard ratio shown for the low-score group therefore represents its mortality hazard relative to the high-score group (** indicates *p* < 0.01, *** indicates *p* < 0.001). (**F**,**G**) Heatmap (**F**) and box plots (**G**) comparing the differential expression patterns of key immune-related genes between the high and low signature score groups (**** indicates *p* < 0.0001). (**H**) Scatter plots quantifying the linear correlation between the signature score and the expression levels of core effector molecules GZMB, PRF1, IFNG, and GNLY. (**I**) Correlation matrix summarizing the Spearman correlation coefficients and statistical significance between the score and various immune molecules (* indicates *p* < 0.05).

Pan-cancer survival analysis showed that the HLA-II-associated CD8^+^ T-cell signature score was significantly associated with overall survival in several malignancies. Higher signature scores were significantly associated with a lower mortality hazard in thyroid carcinoma (THCA), uterine corpus endometrial carcinoma (UCEC), cervical squamous cell carcinoma and endocervical adenocarcinoma (CESC), skin cutaneous melanoma (SKCM), lung adenocarcinoma (LUAD), breast invasive carcinoma (BRCA), sarcoma (SARC), and head and neck squamous cell carcinoma (HNSC), whereas an opposite association was observed in lower-grade glioma (LGG) (Figure 7A). The signature score differed across pan-cancer immune subtypes and was higher in the C2 (IFN-γ-dominant) and C6 (TGF-β-dominant) subtypes (Figure 7B, Kruskal–Wallis *p* < 2.2 × 10^−16^). In contrast, lower scores were observed in the C5 immune subtype, indicating that the signature varies across distinct tumor immune environments (Figure 7B). Pan-cancer correlation analysis showed positive associations between the signature score and cytotoxicity-associated genes, including GZMB, PRF1, and IFNG, as well as dysfunction-associated genes, including PDCD1, LAG3, and TIGIT, in multiple cancer types (Figure 7C).

In the SKCM cohort, Kaplan–Meier analysis showed longer overall survival in the high-score group than in the low-score group (log-rank *p* < 0.0001, Figure 7D). After adjustment for age, sex, and clinical stage, patients in the low-score group had a significantly higher mortality hazard than those in the high-score reference group (HR = 2.00, 95% CI = 1.36–3.00, *p* < 0.001; Figure 7E). The high-score group showed higher expression of multiple effector-associated and immune checkpoint genes than the low-score group (Figure 7F,G). Scatter plot analysis demonstrated a strong positive correlation between the signature score and core effector molecules (Figure 7H), including GZMB (R = 0.86), PRF1 (R = 0.91), and IFNG (R = 0.87). The correlation matrix further showed that the signature score was associated with the expression of multiple genes involved in T-cell activation and cytotoxicity in the SKCM cohort (Figure 7I). Collectively, these results link the HLA-II-associated CD8^+^ T-cell signature to favorable survival and an immune-effector-rich tumor microenvironment in SKCM.

**Figure 8:**
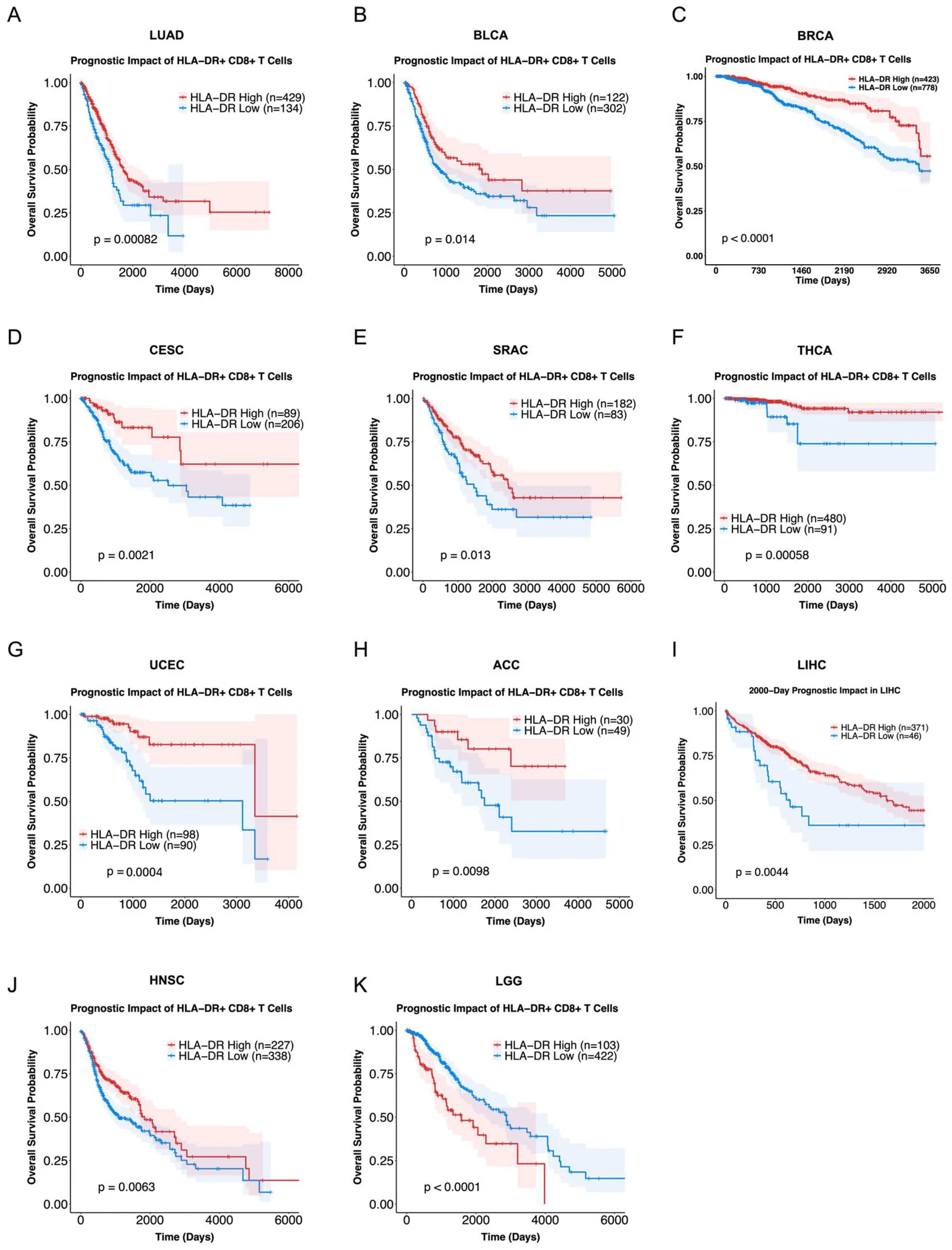
Cancer-Type-Specific Associations Between the HLA-II-Associated CD8^+^ T-Cell Signature and Overall Survival. (**A**–**J**) Cancer cohorts demonstrating a significant prognostic protective effect. Kaplan–Meier analysis was utilized to assess differences in overall survival (OS) within the following cohorts: (**A**) lung adenocarcinoma (LUAD), (**B**) bladder urothelial carcinoma (BLCA), (**C**) breast invasive carcinoma (BRCA), (**D**) cervical squamous cell carcinoma and endocervical adenocarcinoma (CESC), (**E**) sarcoma (SARC), (**F**) thyroid carcinoma (THCA), (**G**) uterine corpus endometrial carcinoma (UCEC), (**H**) adrenocortical carcinoma (ACC), (**I**) liver hepatocellular carcinoma (LIHC), and (**J**) head and neck squamous cell carcinoma (HNSC). The results indicate that patients with a high HLA-DR^+^ CD8^+^ T signature score (red curves) show significantly longer overall survival compared to those in the low-score group (blue curves). (**K**) Reversed prognostic trend in the glioma cohort. In the lower-grade glioma (LGG) cohort, a high signature score is significantly associated with poorer clinical prognosis.

We next examined the association between the HLA-II-associated CD8^+^ T-cell signature and overall survival within individual TCGA cancer cohorts. Kaplan–Meier survival analysis identified nominal associations between higher signature scores and longer overall survival in several cancer cohorts. In LUAD (Figure 8A), BLCA (Figure 8B), BRCA (Figure 8C), CESC (Figure 8D), SARC (Figure 8E), and HNSC (Figure 8J), patients in the high-score group exhibited significantly improved survival compared to those in the low-score group (*p* < 0.05). The strongest statistical evidence for survival differences between the score groups was observed in BRCA and UCEC (log-rank *p* < 0.0001; Figure 8C,G). Furthermore, the signature score demonstrated survival associations in LIHC (Figure 8I) and ACC (Figure 8H) despite varying sample sizes. However, a reversed trend was observed in lower-grade glioma (LGG, Figure 8K), where high scores were associated with poorer prognosis (*p* < 0.0001). In summary, the HLA-II-associated CD8^+^ T-cell signature showed cancer-type-dependent associations with overall survival. Higher scores were associated with longer survival in several cohorts, whereas an opposite association was observed in LGG.

### 2.8. HLA-II-Associated Features Improve the Prediction of Experimentally Annotated Tumor-Reactive TCR Clonotypes

**Figure 9:**
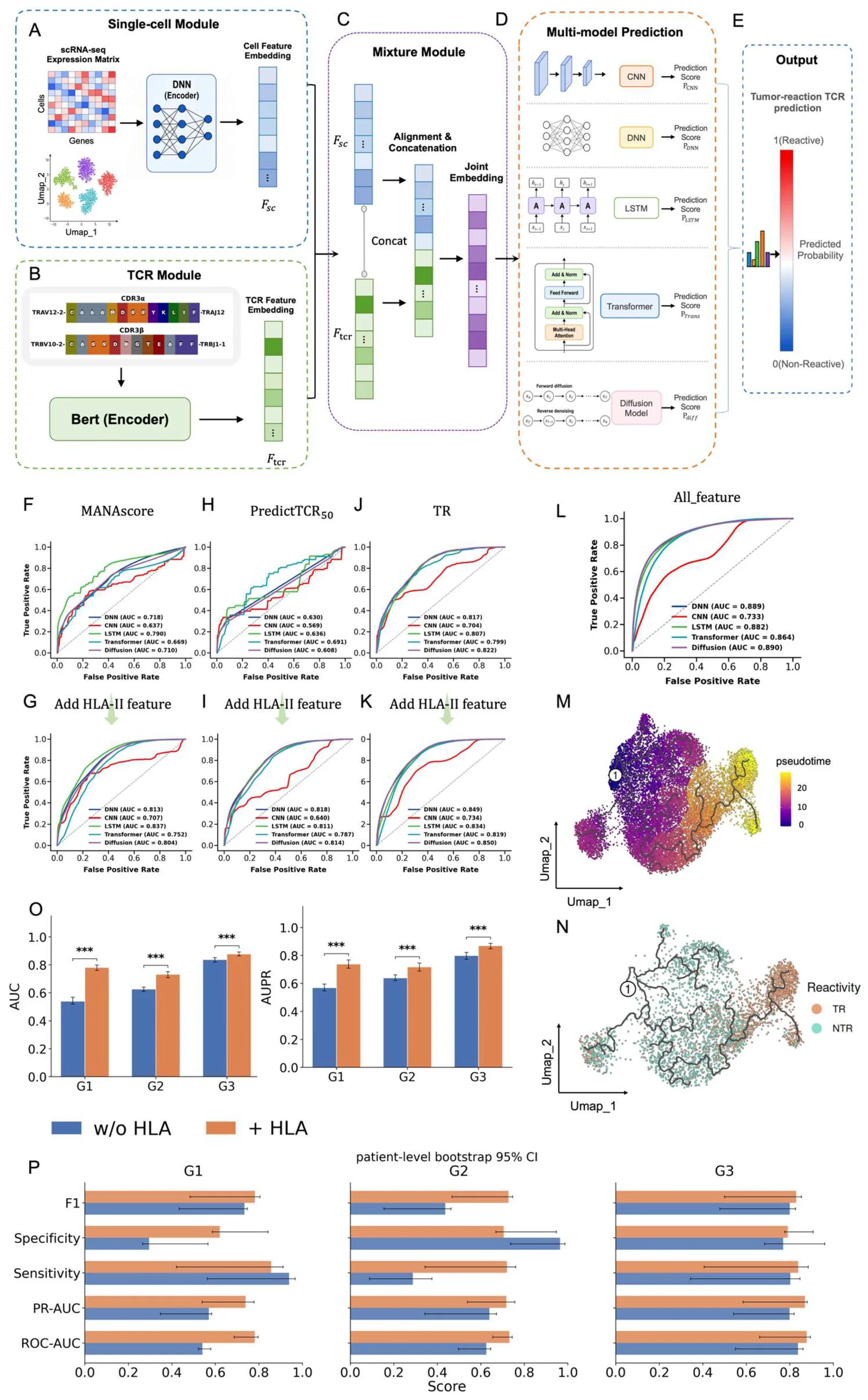
HLA-II-informed multimodal learning and transfer-learning evaluation of experimentally annotated tumor-reactive TCR clonotypes. (**A**–**E**) Schematic of the deep learning model architecture. (**A**) The Single-cell Module utilizes a Deep Neural Network (DNN) encoder to extract cell-state embedding features ($F_{sc}$) from the single-cell gene expression matrix. (**B**) The TCR Module employs a pre-trained BERT model as an encoder to vectorize paired αβ-chain CDR3 sequences and extract sequence embedding features ($F_{tcr}$). (**C**) The Mixture Module generates a joint embedding vector via feature alignment and concatenation. (**D**) CNN, DNN, LSTM, Transformer, and diffusion-based classifiers generate model-specific prediction scores. (**E**) The Output Layer provides a probability score for tumor reactivity. (**F**–**K**) ROC analyses comparing models without and with HLA-II-associated features for the MANAScore (**F**,**G**), PredictTCR_50_ (**H**,**I**), and TR (**J**,**K**) feature sets. Panels F, H, and J show the baseline feature sets, whereas panels G, I, and K show the corresponding feature sets after incorporation of HLA-II-associated genes. (**L**) ROC performance of the models after integration of all available features. (**M**) Pseudotime projection showing the differentiation trajectory of PDAC-infiltrating T cells. The circled number marks the trajectory branch point, and the black lines represent the reconstructed pseudotime trajectory. (**N**) Distribution of experimentally annotated tumor-reactive (TR) and non-tumor-reactive (NTR) cells on the same differentiation manifold. (**O**) Pooled ROC-AUC and PR-AUC comparisons for the G1, G2, and G3 feature panels in 2225 functionally annotated CD8^+^ T cells from the held-out TIPC309, TIPC413, and TIPC416 samples. Models containing the baseline feature panels (w/o HLA) were compared with the corresponding models augmented with HLA-DRA, HLA-DRB1, HLA-DRB5, HLA-DPB1, HLA-DPA1, and HLA-DQA1 (+HLA). Asterisks indicate differences in paired cell-level prediction-score distributions assessed using the Wilcoxon signed-rank test (*** *p* < 0.001). (**P**) Patient-level evaluation of ROC-AUC, PR-AUC, sensitivity, specificity, and F1 score for the G1, G2, and G3 feature panels with and without HLA-II genes. Error bars represent 95% confidence intervals estimated using 1000 patient-level cluster bootstrap replicates. Full point estimates, paired differences, confidence intervals, bootstrap *p* values, and Brier scores are provided in Supplementary Tables S2 and S3.

To evaluate whether HLA-II-associated transcriptional features improve the identification of tumor-reactive T cells, we developed a multimodal learning framework based on scRNA-seq and paired scTCR-seq data collected from 31 large-scale studies (Figure 9A–E). The single-cell module encoded transcriptional features from the scRNA-seq expression matrix (Figure 9A), whereas the TCR module encoded paired TCR sequence features (Figure 9B). The resulting representations were aligned and concatenated to generate an integrated feature space (Figure 9C), from which transcriptional feature panels were selected for subsequent tumor-reactivity modeling. CNN, DNN, LSTM, Transformer, and diffusion-based classifiers were evaluated using the integrated features (Figure 9D), and the output layer generated a probability score for tumor reactivity (Figure 9E). During prediction, the selected gene-expression profile served as the core model input; when paired TCR information was unavailable, predictions were generated using the corresponding scRNA-seq feature profile alone.

We first pretrained the models using clonotype expansion status as the training label. The pretraining dataset comprised 184,207 cells assigned to 98,758 paired TCRαβ clonotypes across 139 samples. The expansion label was assigned at the clonotype level according to the number of cells assigned to each paired TCRαβ clonotype. Clonotypes containing more than 1000 cells were labeled as expanded, whereas those containing 1000 cells or fewer were labeled as non-expanded. The expansion-pretrained model was subsequently adapted using experimentally annotated tumor-reactive and non-reactive TCR clonotypes.

Initial ablation analyses showed that adding HLA-II features improved discrimination across three previously established feature sets and multiple model architectures (Figure 9F–K). In the MANAScore [27] feature set, the DNN AUC increased from 0.718 to 0.813 and the LSTM AUC increased from 0.790 to 0.837 after incorporation of HLA-II features (Figure 9F,G). Comparable improvements were observed for the PredictTCR_50_ [28] feature set (Figure 9H,I) and the TR [29] feature set (Figure 9J,K). When all available features were integrated, the DNN and diffusion models achieved AUCs of 0.889 and 0.890, respectively (Figure 9L).

To adapt the expansion-pretrained model to experimentally defined tumor reactivity, we performed transfer learning using functionally annotated TR and NTR cells from TIPC249, TIPC262, TIPC282, TIPC301, TIPC418, and TIPC432 in GSE254250. TIPC309, TIPC413, and TIPC416 were reserved as independent patient-level test datasets and were not used for model adaptation or model selection. Transcriptomic embedding resolved the PDAC-infiltrating T cells into eight annotated states, including Tn, Tprolif, Trm, Tm, Tex, Temra, Tem and early Tem (Supplementary Figures S8 and S9). Pseudotime analysis delineated the differentiation trajectory of PDAC-infiltrating T cells (Figure 9M), and mapping the experimentally determined functional annotations onto this trajectory revealed the distribution of TR and NTR cells across the corresponding transcriptional states (Figure 9N).

The transfer-learned DNN was evaluated using 2225 functionally annotated CD8^+^ T cells from the three held-out PDAC samples. Three transcriptional feature panels were compared with and without the addition of six HLA-II genes. G1 comprised CXCL13, ENTPD1, and IL7R; G2 comprised CXCL13 and CD40LG; and G3 comprised CTLA4, CCDC141, HAVCR2, GZMB, IL7R, GPR183, SLC46A3, CXCL13, PLEKHA5, LMNA, NKG7, and VCAM1. The HLA-II-augmented versions additionally included HLA-DRA, HLA-DRB1, HLA-DRB5, HLA-DPB1, HLA-DPA1, and HLA-DQA1, resulting in 9, 8, and 18 total genes for G1, G2, and G3, respectively.

In the pooled analysis of the 2225 functionally annotated cells, incorporation of the six HLA-II genes increased both ROC-AUC and PR-AUC across all three feature panels (Figure 9O). The largest improvement was observed for G1, for which ROC-AUC increased from 0.540 to 0.781 (Δ = 0.241) and PR-AUC increased from 0.570 to 0.739 (Δ = 0.169). For G2, ROC-AUC increased from 0.626 to 0.732 and PR-AUC increased from 0.640 to 0.718. G3 already showed comparatively strong baseline performance without HLA-II genes, with an ROC-AUC of 0.838 and a PR-AUC of 0.799; addition of the HLA-II module further increased these values to 0.878 and 0.869, respectively. Paired cell-level Wilcoxon tests detected differences in prediction-score distributions between models with and without HLA-II genes for all three feature panels (*p* < 0.001).

Patient-level cluster bootstrap analysis with 1000 replicates further supported the improvements in discrimination associated with HLA-II augmentation (Figure 9P and Supplementary Tables S2 and S3). The paired increases in ROC-AUC were 0.241, 0.106, and 0.040 for G1, G2, and G3, respectively, whereas the corresponding increases in PR-AUC were 0.168, 0.078, and 0.071 (all bootstrap *p* < 0.001). The threshold-dependent effects varied among feature panels: G1 showed a substantial increase in specificity accompanied by reduced sensitivity, with no significant change in F1 score; G2 showed significant improvements in sensitivity and F1 score despite a nonsignificant numerical reduction in specificity; and G3 showed a modest improvement in F1 score without significant changes in sensitivity or specificity. Brier scores decreased from 0.304 to 0.203 for G1 and from 0.280 to 0.254 for G2, but remained comparable for G3 (0.172 versus 0.175). Full point estimates, confidence intervals, paired differences, and bootstrap *p* values are provided in Supplementary Tables S2 and S3.

## 3. Materials and Methods

### 3.1. Collection, Preprocessing, and Integration of Large-Scale Single-Cell Datasets

The foundational dataset used in this study was assembled from 31 independent single-cell immune-sequencing datasets (Supplementary Figure S1A), encompassing paired single-cell RNA (scRNA-seq) and TCR (scTCR-seq) sequencing libraries. Detailed information, including the accession numbers, for the 31 public datasets included in this study is provided in Supplementary Table S1. All raw sequencing data were processed uniformly using Cell Ranger [30] to obtain gene expression count matrices and TCR contig annotations. During the preliminary quality-control phase, low-quality cells with fewer than 200 detected genes or a mitochondrial gene expression proportion exceeding 20% were excluded. To eliminate potential interference from cell doublets, the DoubletFinder [31] algorithm was employed with default parameters to identify and remove suspected doublets. All included samples were meticulously annotated according to their study source, disease status, and individual identifiers to ensure the traceability of subsequent analyses.

In the process of constructing a high-confidence T-cell reservoir, we performed multimodal integration of gene expression count matrices and TCR clonotype information using the Python-based Scanpy [32] and Scirpy [33] packages. To ensure the precision of the TCR sequence analysis, cells lacking full-length V(D)J sequence information were excluded. Furthermore, to purify the T-cell population and eliminate heterogeneous cell contamination, a marker-based filtering strategy was applied to remove non-target cells expressing B cell markers (MS4A1, CD19) or myeloid markers (LYZ, CD14, CSF3R, and S100A8). This multistage filtering procedure reduced contamination by non-T-cell populations before downstream analyses.

### 3.2. Analysis of T-Cell Subpopulation Composition and Distribution

To characterize the distribution patterns and relative abundances of T-cell subpopulations across different anatomical compartments, including peripheral blood, normal tissues, and tumor tissues, we performed a high-resolution frequency analysis focused on the major lineages of CD4^+^ T and CD8^+^ T cells. To mitigate the effects of inter-individual heterogeneity, the proportion of each cell subtype was calculated independently within each biological sample. The specific calculation method involved normalizing the raw cell count of a particular subtype in each sample to the total number of cells within its corresponding lineage. Specifically, the normalized frequency $f_{i,j,k}$ of subpopulation i within lineage k for sample j is defined as $f_{i,j,k}=\frac{N_{i,j,k}}{\sum_{m=1}^{n}N_{m,j,k}}\times 100\%$, where $N_{i,j,k}$ represents the absolute cell count of the subpopulation and the denominator signifies the aggregate count of all constituent subtypes within the same lineage.

Statistical visualization was conducted using Python (v3.9.23), along with the Scanpy (v1.9.3), Matplotlib (v3.7.1), and Seaborn (v0.12.2) libraries. To enhance biological interpretability, cell subpopulations within each lineage were arranged in descending order according to their mean frequency in the tumor group. In each column of the resulting plots, the Y-axis scales for frequency across the blood, normal tissue, and tumor groups were standardized to ensure that quantitative comparisons of infiltration density remained intuitively comparable. Data are presented as the mean, while error bars represent the standard error of the mean (SEM) derived from independent biological replicates.

### 3.3. Integrated Clonal Analysis via TCR-Based Aggregation and Majority-Voting Annotation

To characterize T-cell states and clonotype-associated transcriptional features across tumor-derived and healthy-control samples, we integrated single-cell transcriptomic (scRNA-seq) and TCR sequencing (scTCR-seq) profiles. T-cell clones were rigorously defined by constructing unique identifiers based on the amino acid sequences of the CDR3 region coupled with specific TRAV, TRAJ, TRBV, and TRBJ gene segments. By aggregating cells sharing identical TCR sequences, we generated a clonal-level dataset to facilitate lineage-specific analysis. To quantify the functional state of these cells, we calculated a T-cell activation score $S_{j}$ for each individual cell, defined as the standardized mean expression of a curated activation gene set, specifically $S_{j}=\frac{1}{\left|G\right|}\sum_{g\in G}z_{g,j}$, where $z_{g,j}$ is the Z-score normalized expression of gene g, ∣G∣ denotes the total number of genes contained in the activation gene set.

Clonal subpopulation annotation was refined using a majority voting strategy to ensure robust lineage assignment; the consensus label $L_{C}$ for clone C was identified as the most frequent cluster assignment among its member cells, denoted by $L_{C}={argmax}_{l\in L}\sum_{i\in ∁}ΙΙ(l_{i}=l)$. In this expression, $L_{C}$ represents the final functional annotation assigned to the entire TCR clone C, while L denotes the complete set of all potential subpopulation labels identified during the initial clustering of the single-cell transcriptomic data. The term $l_{i}$ refers to the specific cluster label assigned to an individual cell i within the clone C. To rigorously quantify the frequency of each label, we utilize the indicator function II, which yields a value of 1 if the condition $l_{i}=l$ is satisfied and 0 otherwise. By applying the argmax operator, the strategy effectively selects the label l that maximizes the summation of these indicators, thereby ensuring that the clonal identity reflects the dominant transcriptomic state of the cellular population.

For one-vs-rest analysis of TCR gene-segment usage, each unique clonotype was assigned to a single T-cell subset according to the majority transcriptional annotation of its constituent cells. For each target subset, clonotypes assigned to that subset were compared with clonotypes assigned to all remaining T-cell subsets. Gene-segment usage frequency was defined as the proportion of unique clonotypes containing a given TRAV, TRAJ, TRBV, or TRBJ segment. For each segment, enrichment was evaluated using a two-sided Fisher’s exact test based on the numbers of clonotypes with and without the segment in the target and remaining populations. *p* values were adjusted for multiple testing using the Benjamini–Hochberg method. Effect sizes were expressed as the log_2_ ratio of the gene-segment usage frequency in the target subset to that in the remaining T-cell subsets.

Differential gene expression (DEG) analysis was subsequently performed using the Wilcoxon rank-sum test to pinpoint transcriptomic divergences between tumor-infiltrating and healthy tissue-derived clones. Clonal expansion status was evaluated using the Scirpy framework, with clonotypes represented by more than 1000 cells within an individual biological sample categorized as highly expanded. This integrative pipeline links phenotypic landscapes to specific TCR V/J gene usage, effectively bridging clonal expansion with distinct genetic fingerprints.

### 3.4. CD8^+^ T-Cell Differentiation-State Trajectory Analysis

To characterize the differentiation-state trajectory of CD8^+^ T cells within the tumor microenvironment and its association with HLA-II gene expression, we performed pseudotime trajectory analysis using Monocle2 [34]. Initially, a differentiation trajectory tree was constructed in a low-dimensional space via the Discriminative Dimensionality Reduction with Trees (DDRTree) algorithm. The origin of the pseudotime trajectory was defined as the region enriched with naïve T cells (CD8^+^ Tn). Subsequently, we quantified the progression of cells from their primordial state toward effector and exhausted states by calculating pseudotime values.

Generalized linear models with spline terms were used to fit smoothed expression trends over pseudotime for the HLA-II genes HLA-DPA1, HLA-DPB1, HLA-DQA1, HLA-DQB1, HLA-DRA, HLA-DRB1, and HLA-DRB5 and the cytotoxicity- and effector-associated genes CCL4, GZMB, GZMH, GZMK, NKG7, and PRF1. Cell-subset annotations and HLA-II expression status were subsequently mapped onto the inferred trajectory to characterize their distributions across pseudotime states and branches.

### 3.5. Intercellular T-Cell Communication Landscape Analysis

CellChat [35] was used to infer ligand–receptor communication networks in tumor and normal T-cell datasets. The tumor dataset contained 603,708 T cells from 335 biological samples and 139 donors, whereas the normal-tissue dataset contained 254,709 T cells from 110 biological samples and 51 donors. Direct comparisons were restricted to T-cell subsets represented in both conditions. To reduce cell-number imbalance, cells were stratified according to T-cell subset, and the condition containing more cells within each shared subset was randomly downsampled to the number observed in the smaller condition. The resulting cell-number-balanced datasets were analyzed separately using the same CellChat database, preprocessing steps, communication-probability parameters, and filtering thresholds.

Communication networks were compared according to the predicted number and aggregate strength of interactions. MHC-II pathway-specific networks, sender–receiver patterns, and the relative contributions of individual ligand–receptor pairs were subsequently extracted. Communication probabilities and their statistical significance were estimated using CellChat permutation testing. Network diagrams, sender–receiver heatmaps, ligand–receptor contribution plots, gene-expression distributions, and interaction-probability dot plots were used to compare the inferred communication patterns between tumor and normal tissues.

### 3.6. TCR Clonal Expansion Analysis and HLA-II Expression Scoring

Clonotypes were identified through the integration of single-cell transcriptomics and TCR sequencing data based on CDR3α and CDR3β sequences along with their respective V/J gene annotations. We categorized clones containing more than 1000 member cells as highly expanded and performed a quantitative assessment of clonal distribution across both tumor and paired normal tissues. To quantify the transcriptional activity of HLA class II molecules, we selected a set of classical HLA-II genes including HLA-DRA, HLA-DPA1, and HLA-DPB1 to calculate an average Z-score for each cell, which served as the “HLA-II Expression Score.” Statistical differences in scores across various expansion states and tissue origins were evaluated using the Wilcoxon rank-sum test.

### 3.7. In Vitro T-Cell Activation, Proliferation, and HLA-DR Blockade Assays

Cryopreserved primary human peripheral blood mononuclear cells (PBMCs) derived from healthy donors were purchased from MileCell Bio (Shanghai, China; Cat. No. PB010F-C). After thawing, the cells were washed and recovered in complete culture medium according to the supplier’s instructions. Untouched CD8^+^ T cells were subsequently isolated from the PBMCs by negative selection using the CD8^+^ T-Cell Isolation Kit, human (Miltenyi Biotec, Bergisch Gladbach, Germany; Cat. No. 130-096-495), according to the manufacturer’s instructions. The purity of the isolated CD8^+^ T-cell population was assessed by flow cytometry before the activation and proliferation experiments. A representative purification result is shown in Supplementary Figure S3.

PBMCs were seeded into 24-well plates at a density of $1\times 10^{5}$ cells per well in 1 mL of RPMI 1640 medium supplemented with 10% fetal bovine serum (FBS) and 1% penicillin-streptomycin. Purified CD8^+^ T cells were seeded at $1\times 10^{5}$ cells per well under the same culture conditions. Three parallel treatment groups were established for both the PBMC and purified CD8^+^ T-cell experiments: (1) an unstimulated control group maintained in complete medium without exogenous stimulation; (2) a stimulation group treated with Dynabeads Human T-Activator CD3/CD28 (Thermo Fisher Scientific, Waltham, MA, USA; Cat. No. 11161D) at a 1:1 bead-to-cell ratio together with recombinant human IL-2 at 200 IU/mL; and (3) an HLA-DR-blockade group receiving the same CD3/CD28 and IL-2 stimulation together with 10 μg/mL of the HLA-DR-blocking monoclonal antibody L243 (BioLegend, San Diego, CA, USA; Cat. No. 307602). All cultures were maintained at 37 °C in a humidified atmosphere containing 5% CO_2_.

For analysis of CD25 expression, PBMC and purified CD8^+^ T-cell cultures were harvested after 72 h. For analysis of CD69 expression, both PBMC and purified CD8^+^ T-cell cultures were harvested after 72 h. Cells were stained with PE-conjugated anti-human CD25 antibody (Proteintech, Rosemont, IL, USA; Cat. No. PE-65096), PE-Cyanine7-conjugated anti-human CD8 antibody, clone UCHT4 (Proteintech; Cat. No. PY7-65559), and FITC Plus-conjugated anti-human CD69 antibody (Proteintech; Cat. No. FITC-98173). CD25 and CD69 were assessed in separate staining panels. Flow-cytometry data were acquired using a BD Accuri C6 Plus flow cytometer (BD Biosciences, San Jose, CA, USA) and analyzed using FlowJo software version 10.9.0.

Fluorescence compensation was applied before gating using a compensation matrix generated from single-stained controls for each fluorochrome. Positive gates for CD25 and CD69 were established using unstained controls. The sequential gating strategy comprised the identification of lymphocytes using FSC-A versus SSC-A, exclusion of doublets and aggregates using FSC-A versus FSC-H, selection of CD8^+^ cells, and assessment of CD25 or CD69 positivity within the gated CD8^+^ singlet lymphocyte population. Percentages displayed beside the gates were calculated relative to the immediate parent population. The same gating hierarchy was applied to all experimental groups and biological replicates. A representative complete gating hierarchy is provided in Supplementary Figure S4.

For proliferation analysis, purified CD8^+^ T cells were labeled before stimulation using the CellTrace^TM^ CFSE Cell Proliferation Kit (Thermo Fisher Scientific; Cat. No. C34570). Cells were resuspended in 5 μmol/L CellTrace CFSE staining solution, incubated at 37 °C for 20 min while protected from light, and washed with complete culture medium to remove excess dye. The labeled cells were subsequently cultured under the three treatment conditions described above for 5 days. CellTrace^TM^ CFSE dilution was measured by flow cytometry. Proliferating cells were identified within the gated CD8^+^ singlet population using the CFSE-labeled unstimulated group as the reference for undivided cells. Proliferation was expressed as the percentage of CFSE-low divided CD8^+^ T cells.

All activation and proliferation experiments were independently repeated three times. Results are presented as the mean ± standard error of the mean (SEM). Differences among the three treatment groups were evaluated using one-way analysis of variance (ANOVA) followed by Tukey’s multiple-comparison test. *p* < 0.05 was considered statistically significant, and **** indicates *p* < 0.0001.

### 3.8. Analysis of Amino Acid Usage Within CDR3 Junctional Regions

To precisely characterize the recombination features of TCR sequences, we defined the central junctional sequences of the CDR3 regions as CDR3α-mr and CDR3β-mr. These sequences represent the amino acid segments encoded by stochastic nucleotide insertions between the V and J gene segments. By removing the conserved amino acids derived from the germline V and J loci, we extracted and annotated the CDR3-mr residues generated exclusively through junctional diversity.

On this basis, we quantified the distribution of physicochemical properties across T-cell subpopulations by calculating the usage frequency of amino acids with different chemical characteristics within each TCR sequence. The mean values were then compared across groups to identify specific preferences. To evaluate the statistical significance of these feature differences between subpopulations, we applied a paired two-tailed Student’s *t*-test for the single-cell TCR sequencing (scTCR-seq) data and a standard two-tailed Student’s *t*-test for the bulk TCR sequencing data. This stratified statistical strategy ensures the robustness and reliability of the physicochemical preference analysis across different data scales.

### 3.9. Pan-Cancer Prognostic Analysis and Immune Signature Modeling

To evaluate the pan-cancer clinical associations of the HLA-II-associated CD8^+^ T-cell signature, we obtained transcriptomic data and corresponding clinical follow-up information for 33 common cancer types from The Cancer Genome Atlas (TCGA) database. A signature score for this cell population was constructed based on HLA-DR-related genes and CD8^+^ T-cell markers. We then assessed the predictive efficacy of this score for overall survival (OS) across various cancer types using univariate Cox proportional hazards regression analysis, from which hazard ratios (HRs) and 95% confidence intervals were calculated.

To compare the distribution of the signature score across the six established pan-cancer immune subtypes (C1–C6), the Kruskal–Wallis test was employed. Furthermore, Spearman correlation analysis was used to calculate the strength of the association between the signature score and classical immune effector molecules as well as checkpoint genes, including GZMB, PRF1, IFNG, PDCD1, and LAG3, at both the pan-cancer level and within specific cancer types.

### 3.10. Clinical Validation and Statistical Modeling in Independent Cancer Cohorts

For the skin cutaneous melanoma (SKCM) cohort, we utilized the Kaplan–Meier method to generate survival curves and performed log-rank tests to analyze prognostic differences between high-score and low-score groups. To verify the independent prognostic value of the signature score, we constructed a multivariate Cox regression model incorporating key clinical covariates including age, sex, and clinical stage. Subsequently, the differential expression patterns of immune-related genes between the distinct scoring groups were visualized through heatmaps and box plots. Finally, scatter plots and correlation matrices were employed to precisely quantify the linear correlation between the HLA-DR^+^ CD8^+^ T score and the expression levels of cytotoxic effectors and immune checkpoint genes within the SKCM cohort.

To further validate the prognostic stability of the HLA-DR^+^ CD8^+^ T-cell signature score across diverse biological contexts, we conducted individual survival analyses for 11 major cancer types in TCGA including LUAD, BLCA, BRCA, CESC, SARC, THCA, UCEC, ACC, LIHC, HNSC, and LGG. Patients were stratified into high and low signature score groups using the median score as the cut-off value. Overall survival (OS) curves were plotted via the Kaplan–Meier method, and the statistical significance of survival differences between groups was evaluated using the log-rank test.

### 3.11. Construction and Pretraining of the Multimodal Deep Learning Framework

The integrated scRNA-seq and paired scTCR-seq datasets described in Section 3.1 were used to construct the model inputs. The scRNA-seq input consisted of cell-level gene-expression profiles, whereas the scTCR-seq input consisted of paired CDR3α and CDR3β sequences when available. Transcriptional and TCR sequence features were encoded separately and subsequently aligned and concatenated to generate an integrated representation. Candidate gene-expression panels derived from the integrated analysis were used as the core inputs for downstream prediction, with TCR-derived embeddings incorporated when paired sequence information was available.

For expansion-based pretraining, the label was assigned at the paired TCRαβ clonotype level. Clonotype size was defined as the number of cells assigned to each clonotype in the pretraining dataset. Clonotypes containing more than 1000 cells were labeled as expanded, whereas those containing 1000 cells or fewer were labeled as non-expanded. The pretraining dataset contained 184,207 cells assigned to 98,758 paired TCRαβ clonotypes across 139 samples.

Gene-expression features were converted to numeric values before model fitting. If a gene was present in the input matrix but its value for an individual cell was missing or non-numeric, the corresponding value was replaced with 0. Genes that were entirely absent from a target dataset were not assigned artificial expression values; prediction panels were restricted to genes available in both the training and target datasets. Expression features were standardized using parameters estimated exclusively from the corresponding training partition and subsequently applied without re-estimation to validation and test data.

CDR3α and CDR3β sequences were parsed from the paired TCR annotations, and invalid amino-acid characters were removed. When paired TCR sequence information was unavailable, the TCR encoding branch was not invoked and prediction was based on the selected scRNA-seq feature profile.

The gene-expression branch of the DNN consisted of two fully connected hidden layers containing 256 and 128 units, respectively. Each hidden layer was followed by ReLU activation and dropout with a rate of 0.2, and the output layer contained a single unit. The model was optimized using Adam with a learning rate of 1 × 10^−3^ and binary cross-entropy with logits. Class imbalance was addressed using a weighted random sampler and a positive-class weight calculated as the ratio of negative to positive observations and capped at 20. Models were trained for 20 epochs. CNN, LSTM, Transformer, and diffusion-based classifiers were evaluated using the same input feature panels.

### 3.12. Grouped Cross-Validation, Transfer Learning, and Patient-Level Evaluation

Internal model performance was evaluated using five-fold sample-grouped cross-validation. The sample identifier was used as the grouping variable, ensuring that all cells from the same sample were assigned to the same fold and that no sample was shared between the training and validation partitions. In each iteration, four folds were used for model training and the remaining fold was used for validation. Predictions from the held-out fold were retained as out-of-fold predictions and combined across the five iterations for performance evaluation. Thus, model performance was not assessed using a random cell-level split. The multi-cancer pretraining data were pooled across studies and cancer types and were not partitioned according to study or cancer type. A single prespecified random seed of 42 was used throughout model training and other randomized procedures.

The expansion-pretrained DNN was subsequently adapted to experimentally defined tumor reactivity using GSE254250. Functionally annotated TR and NTR cells from TIPC249, TIPC262, TIPC282, TIPC301, TIPC418, and TIPC432 were used for transfer learning. TIPC309, TIPC413, and TIPC416 were retained as independent sample-level test datasets. No cells from these three samples were used for transfer learning, hyperparameter optimization, feature selection, or model selection.

Three gene-expression panels were evaluated in the held-out test datasets. G1 contained CXCL13, ENTPD1, and IL7R; G2 contained CXCL13 and CD40LG; and G3 contained CTLA4, CCDC141, HAVCR2, GZMB, IL7R, GPR183, SLC46A3, CXCL13, PLEKHA5, LMNA, NKG7, and VCAM1. For each panel, the HLA-II-augmented model additionally included HLA-DRA, HLA-DRB1, HLA-DRB5, HLA-DPB1, HLA-DPA1, and HLA-DQA1, resulting in 3 versus 9 genes for G1, 2 versus 8 genes for G2, and 12 versus 18 genes for G3.

Model performance was assessed using ROC-AUC, PR-AUC, sensitivity, specificity, F1 score, and Brier score. Calibration was evaluated using Brier scores and calibration curves. For the held-out PDAC evaluation, uncertainty was quantified using 1000 patient-level cluster bootstrap replicates. In each replicate, samples rather than individual cells were resampled with replacement, and all functionally annotated cells from each selected sample were retained. Models with and without HLA-II genes were evaluated using the same bootstrap samples, thereby preserving the paired comparison. The 2.5th and 97.5th percentiles of the bootstrap distributions were used to derive 95% confidence intervals.

Paired differences in ROC-AUC, PR-AUC, sensitivity, specificity, and F1 score were calculated within each bootstrap replicate by subtracting the performance of the model without HLA-II genes from that of the corresponding HLA-II-augmented model. Two-sided bootstrap *p* values were estimated from the proportions of paired differences on either side of zero. Detailed point estimates, confidence intervals, paired differences, and bootstrap *p* values are provided in Supplementary Tables S2 and S3.

### 3.13. Functional Annotation and Trajectory Analysis of the PDAC Dataset

The GSE254250 dataset [24] was used to relate the model predictions to experimentally determined TCR reactivity in pancreatic ductal adenocarcinoma (PDAC). In the original study, selected TCR clonotypes were functionally evaluated after TCR mRNA transfer followed by coculture with autologous tumor cells, with tumor reactivity determined using TNF-α production and/or CD107a mobilization. Cells assigned to experimentally validated tumor-reactive clonotypes were labeled as tumor-reactive (TR), whereas cells assigned to experimentally tested non-reactive clonotypes were labeled as non-tumor-reactive (NTR). Cells without an experimental TR or NTR annotation were excluded from model-performance evaluation. The allocation of the functionally annotated samples to the transfer-learning and held-out test datasets is described in Section 3.12.

PDAC T cells that passed quality-control filtering were projected into a UMAP-based low-dimensional transcriptional manifold and annotated according to their transcriptional states (Supplementary Figure S9). Pseudotime inference was performed using Monocle3 [36], and a principal graph was learned on the reduced-dimensional representation to characterize the distribution of cells along the inferred differentiation-state trajectory. Experimentally determined TR and NTR annotations were subsequently mapped onto the same manifold to examine their distributions across transcriptional and differentiation states. The resulting pseudotime projection and functional-label distribution are shown in Figure 9M and Figure 9N, respectively.

A complete list of the principal software packages, computational environments, and corresponding versions used in this study is provided in Supplementary Table S4.

## 4. Conclusions

In this study, we integrated pan-cancer single-cell transcriptomic and TCR repertoire data to characterize transcriptional programs associated with tumor-reactive CD8^+^ T cells. Across multiple tumor types, we identified a distinct HLA-II^+^ CD8^+^ T-cell population enriched for clonally expanded TCRs, cytotoxic signatures, and terminal differentiation states. These findings suggest that HLA-II expression represents a conserved feature associated with sustained antigen-driven activation within the tumor microenvironment.

Although HLA class II molecules are classically restricted to professional antigen-presenting cells, increasing evidence indicates that activated T cells can ectopically express HLA-II genes under inflammatory conditions. Our results extend these observations by demonstrating that HLA-II expression is tightly associated with clonal expansion and cytotoxic differentiation across diverse cancers. Pseudotime analysis further showed that HLA-II induction emerges during late stages of CD8^+^ T-cell differentiation, coinciding with the acquisition of effector programs such as GZMB, PRF1, and NKG7 expression. These observations support the possibility that HLA-II expression reflects a sustained activation state associated with chronic antigen exposure.

Cell–cell communication analysis additionally revealed enhanced MHC-II signaling interactions between HLA-II^+^ CD8^+^ T cells and CD4^+^ T-cell populations within tumors. Such interactions were largely absent in normal tissues, suggesting the presence of a tumor-associated immune communication program. One potential explanation is that HLA-II^+^ CD8^+^ T cells partially acquire antigen-presentation-related functions that facilitate local immune coordination within the tumor microenvironment. Whether these interactions directly contribute to the maintenance of anti-tumor immunity or instead reflect chronic inflammatory remodeling requires further experimental investigation.

Our functional experiments further support a role for HLA-DR in maintaining activated CD8^+^ T-cell responses. In mixed PBMC cultures, L243 reduced CD25 upregulation while CD69 induction remained high. The reduction in CD25 expression was reproduced in purified CD8^+^ T-cell cultures, and CellTrace CFSE analysis further showed attenuated proliferation following HLA-DR blockade. The concordant effects observed in the enriched CD8^+^ T-cell preparation make it less likely that the findings can be explained solely by accessory-cell populations in mixed PBMC cultures.

From a translational perspective, the HLA-II^+^ CD8^+^ T-cell signature demonstrated broad prognostic relevance across multiple solid tumors and correlated strongly with cytotoxic and exhaustion-associated programs. Importantly, the incorporation of HLA-II-associated features consistently improved prediction performance in our multimodal deep learning framework across several independent datasets. These results suggest that HLA-II expression captures biologically meaningful information relevant to tumor-reactive T-cell identification.

The study also has several limitations. First, most functional conclusions were inferred computationally and require additional mechanistic validation in vivo. Second, the precise molecular mechanisms regulating HLA-II induction in CD8^+^ T cells remain incompletely understood. Future studies integrating epigenetic profiling, spatial transcriptomics, and functional perturbation experiments may further clarify the developmental and regulatory basis of this population.

In summary, our study identifies HLA-II expression as a conserved transcriptional feature associated with tumor-reactive CD8^+^ T cells across cancers. By combining large-scale single-cell analysis with multimodal deep learning, we provide both biological insight into tumor-reactive T-cell states and a computational framework for prioritizing candidate TCRs for precision immunotherapy.

## Data Availability

The original contributions presented in this study are included in the article/Supplementary Material. Further inquiries can be directed to the corresponding authors. The source code and analysis scripts are available on GitHub (https://github.com/Luckysoutheast/TR-HLAII, accessed on 15 January 2026).

## Supplementary Materials

The following supporting information can be downloaded at: https://www.mdpi.com/article/10.3390/ijms27188338/s1.
