# Supplementary material for: HLA-II Expression Marks Activated and Clonally Expanded CD8+ T Cells and Improves Tumor-Reactivity Prediction Across Human Cancers
Source: Int J Mol Sci. 2026 Sep 19;27(18):8338. doi: 10.3390/ijms27188338 (PMC13607992; doi:10.3390/ijms27188338)
Supplement: Supplementary file 1 [file ijms-27-08338-s001.zip › Supplementary Figures.pdf]

Supplementary Figures

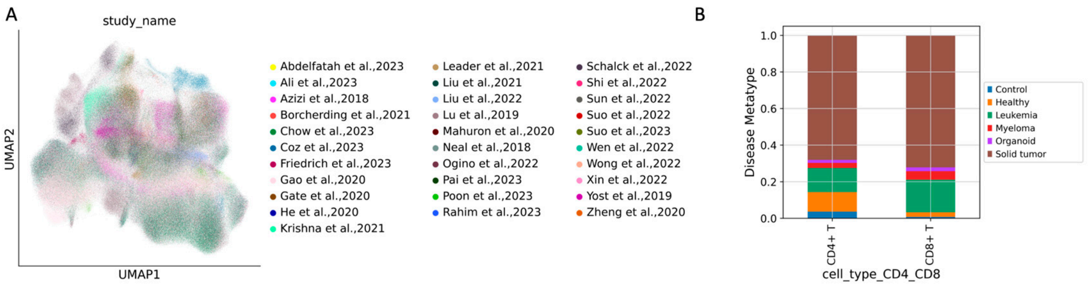

Figure S1. Dataset Integration and Meta-information Distribution. (A) UMAP projection of T cells annotated by study source (Study Name). The plot illustrates the integrated distribution of single-cell transcriptomic data from 31 independent studies within the manifold space. Each color represents an original research dataset, and the results demonstrate that various subpopulations are well-mixed in the embedding space, indicating that major study-associated batch effects were reduced during the integration process. (B) Distribution proportions of disease categories across different T-cell lineages. The stacked bar charts display the distribution of $CD4^+$ T and $CD8^+$ T cells under various disease states, including solid tumors (which represent the dominant fraction), healthy controls, leukemia, myeloma, and organoids. These distributions indicate that this study primarily focuses on the remodeling of T cell function within the solid tumor microenvironment.

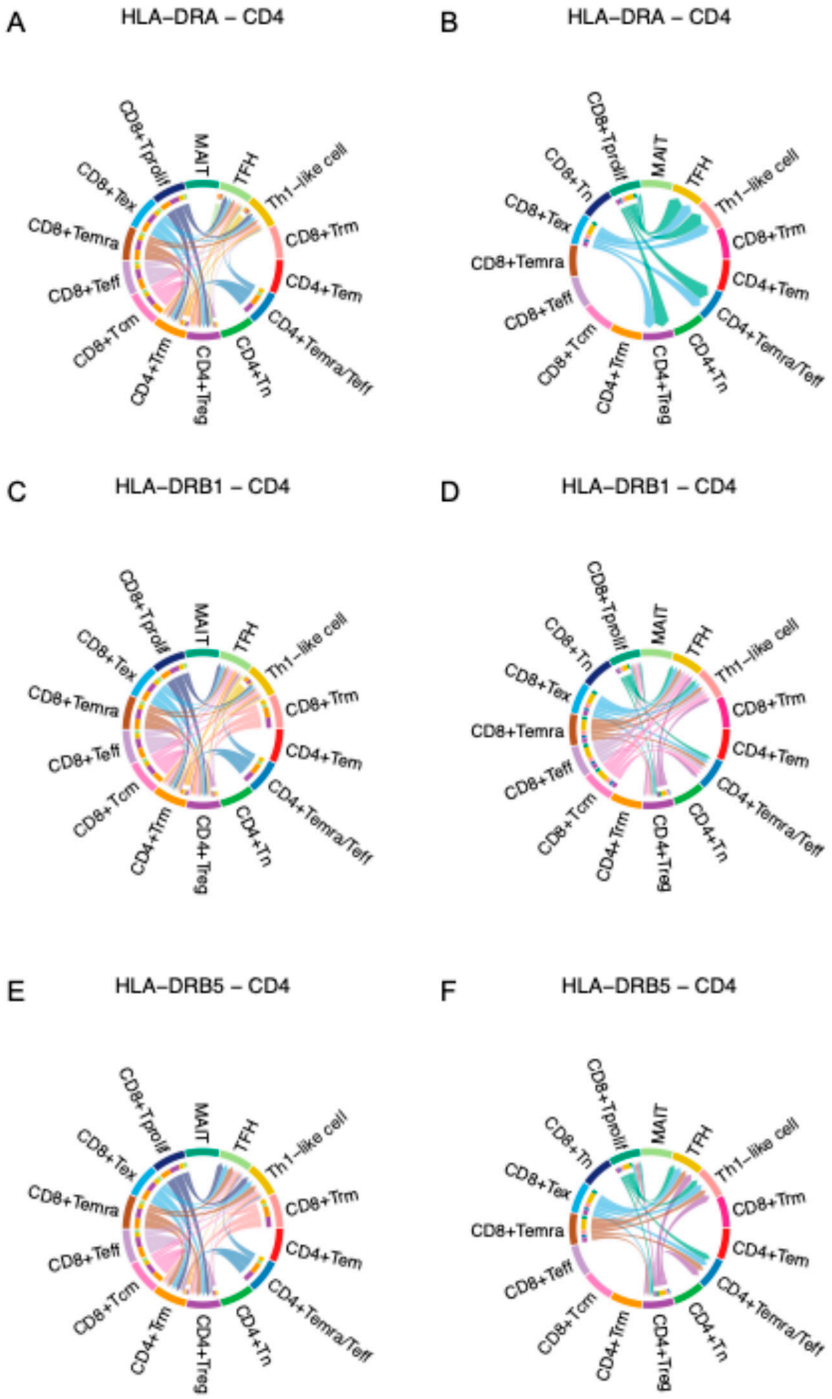

Figure S2. Chord Diagram Analysis of MHC-II Signaling Subsystems. (A, C, E) Chord diagram analysis of MHC-II signaling subsystems in tumor tissues. The diagrams illustrate the interactions of the (A) HLA-DRA/CD4, (C) HLA-DRB1/CD4, and (E) HLA-DRB5/CD4 ligand-receptor pairs among T-cell subpopulations. The thickness of the connecting lines represents communication intensity, and arrows indicate the signal receivers. (B, D, F) Corresponding chord diagram analysis of MHC-II signaling subsystems in healthy tissues. The plots display the communication landscapes of (B) HLA-DRA/CD4,

(D) HLA-DRB1/CD4, and (F) HLA-DRB5/CD4 under healthy conditions, highlighting the comparative lack of signaling activity.

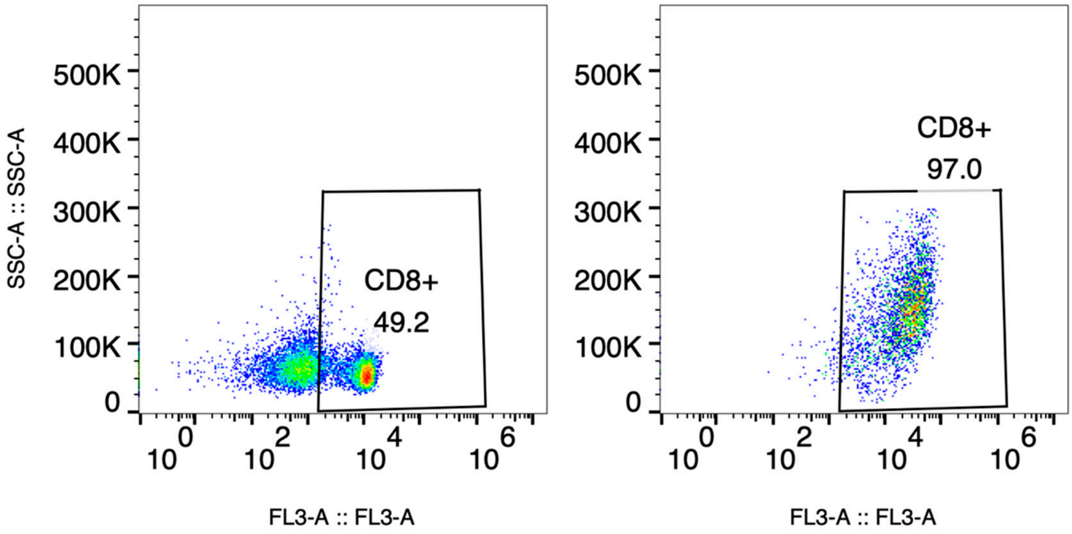

Figure S3. Flow-cytometric assessment of CD8+ T-cell enrichment following negative-selection isolation. Representative flow-cytometry plots showing the percentage of CD8+ cells before isolation (left) and after negative selection using the CD8+ T Cell Isolation Kit, human (Miltenyi Biotec; Cat. No. 130-096-495) (right). CD8+ cells increased from 49.2% to 97.0% of the gated singlet lymphocyte population after isolation. The enriched CD8+ T cells were used in the experiments shown in Figure 5C–F.

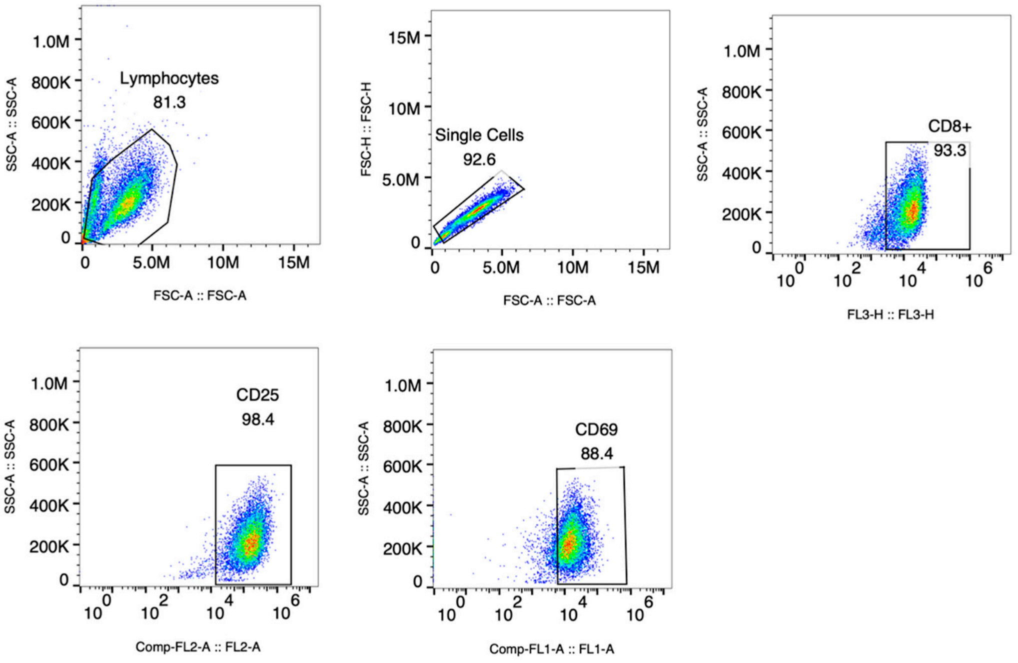

Figure S4. Representative Flow-Cytometry Gating Strategy for CD8^{+} Lymphocyte Activation-

Marker Analysis. Lymphocytes were identified using FSC-A versus SSC-A, followed by selection of single cells using FSC-A versus FSC-H. CD8+ cells were subsequently identified within the singlet lymphocyte population, and CD25 and CD69 positivity were assessed separately within the gated CD8+ singlet population. Fluorescence compensation was applied before gating using single-stained controls, and positive gates were established using unstained controls. Values displayed beside each gate indicate the percentage of events relative to the immediate parent population. The same gating hierarchy was applied to all treatment groups and biological replicates. Data were analyzed using FlowJo version 10.9.0.

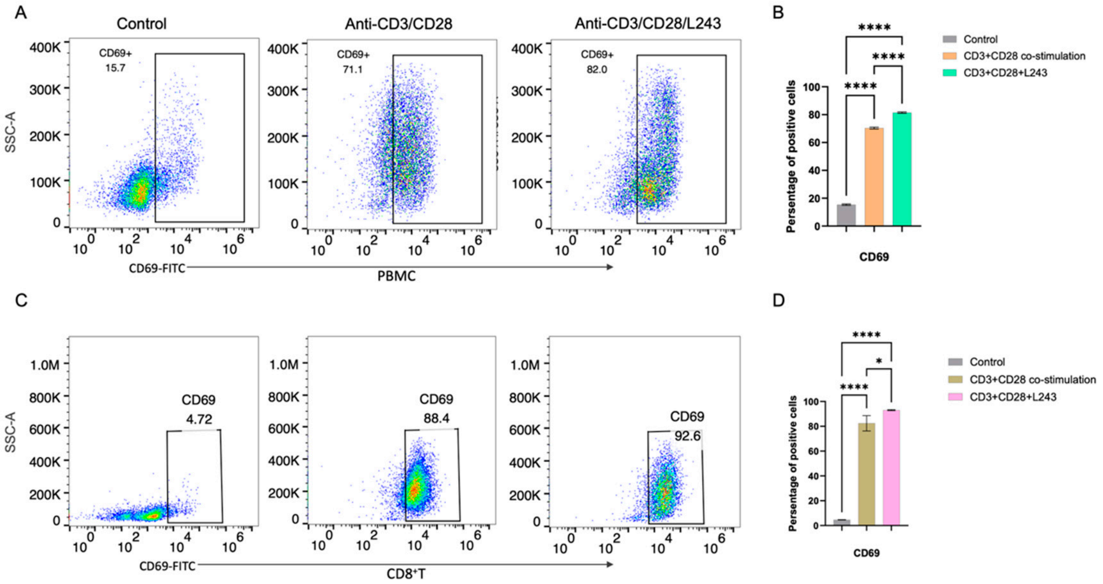

Figure S5. CD69 expression following HLA-DR blockade in mixed PBMC and purified CD8+ T-cell cultures. (A) Representative flow-cytometry plots showing CD69 expression within gated CD8+ singlet lymphocytes under unstimulated conditions, following anti-CD3/CD28 plus IL-2 stimulation, or following the same stimulation in the presence of the HLA-DR-blocking antibody L243. Values shown beside the gates indicate the percentage of CD69+ cells within the gated CD8+ singlet lymphocyte population. (B) Quantification of the percentage of CD69+ cells across the three treatment groups. Data are presented as the mean ± SEM from three independent experiments. (C) Representative flow-cytometry plots showing CD69 expression in purified CD8+ T-cell cultures under the same three treatment conditions. (D) Quantification of the percentage of CD69+ cells within the gated purified CD8+ singlet population. Bars represent the mean, and error bars indicate the SEM from three independent experiments. Statistical significance was evaluated using one-way ANOVA followed by Tukey's multiple-comparison test (**** indicates $P < 0.0001$, * indicates $P < 0.05$).

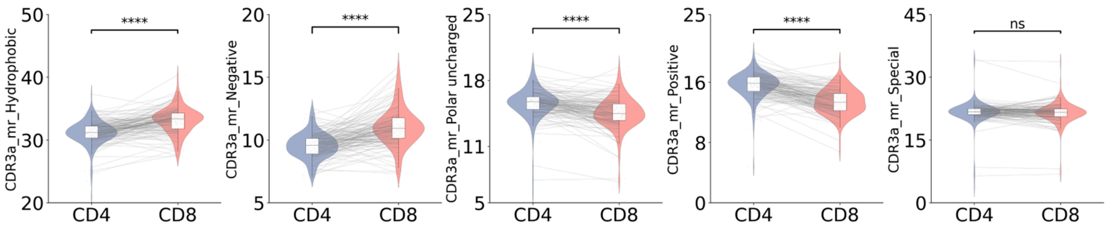

Figure S6. Distribution of Physicochemical Parameters in the CDR3α-mr Regions of CD4⁺ and CD8⁺ T Cells. The violin plots illustrate the distributional differences in multiple physicochemical parameters within the CDR3α-mr regions of CD4⁺ (blue) and CD8⁺ (red) T cells. The analyzed metrics include the hydrophobicity index, the proportion of negatively charged residues, the proportion of polar uncharged residues, the proportion of positively charged residues, and the proportion of special amino acids. Gray lines connect the distributions of identical clones or corresponding samples to highlight paired differences between the groups (**** indicates $P < 0.0001$, ns indicates $P > 0.05$).

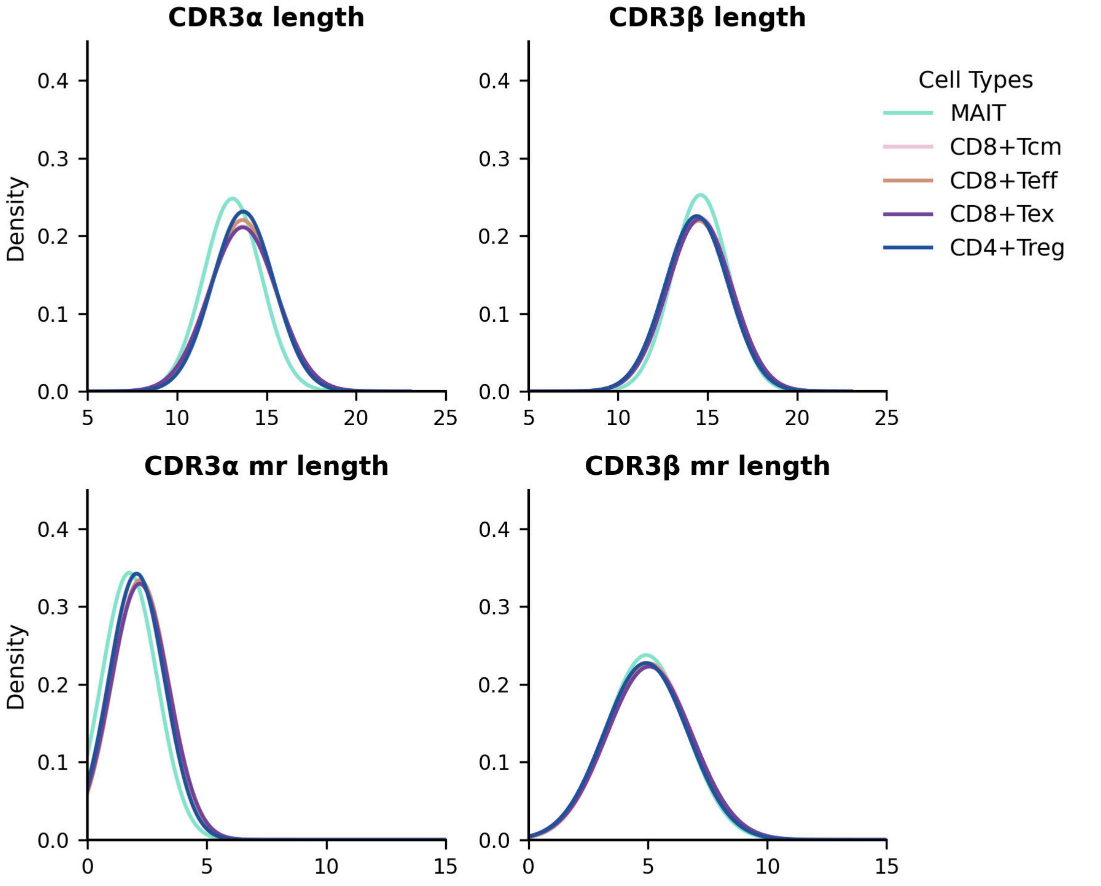

Figure S7. Distribution of Amino Acid Lengths within Key TCR Regions Across T-cell Subpopulations. The density plots illustrate the amino acid length distributions within critical TCR regions across various functional T-cell subpopulations. Statistical analysis encompasses the lengths of the CDR3α, CDR3β, CDR3α-mr, and CDR3β-mr regions. Different colors represent distinct functional subsets,

including MAIT cells (cyan), CD8⁺ Tcm (pink), CD8⁺ Teff (brown), CD8⁺ Tex (dark purple), and CD4⁺ Treg (blue).

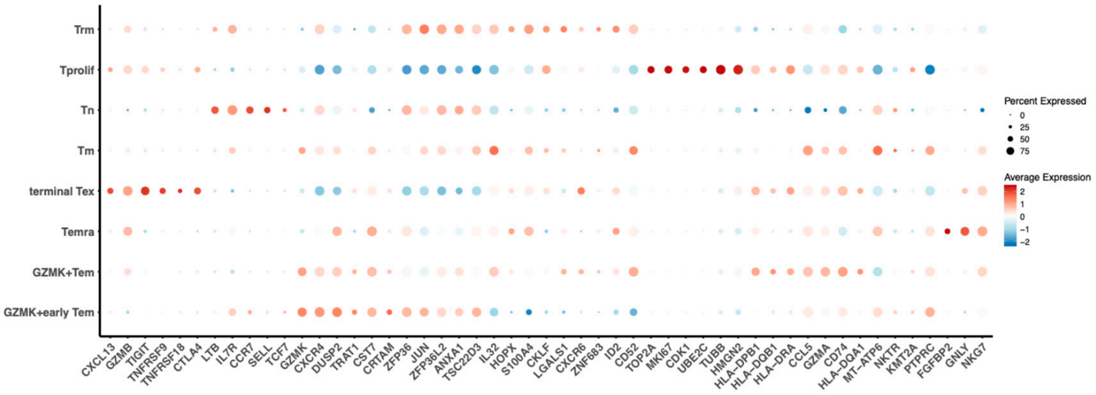

Figure S8. Expression Profiles of Marker Genes Across T-cell Subpopulations in the Pancreatic Ductal Adenocarcinoma (PDAC) Dataset. The bubble plot illustrates the expression profiles of marker genes for various T-cell subpopulations identified within the PDAC dataset. The size of each bubble represents the percentage of cells expressing the gene in a specific subpopulation (Percent Expressed), and the color intensity indicates the average expression level (Average Expression).

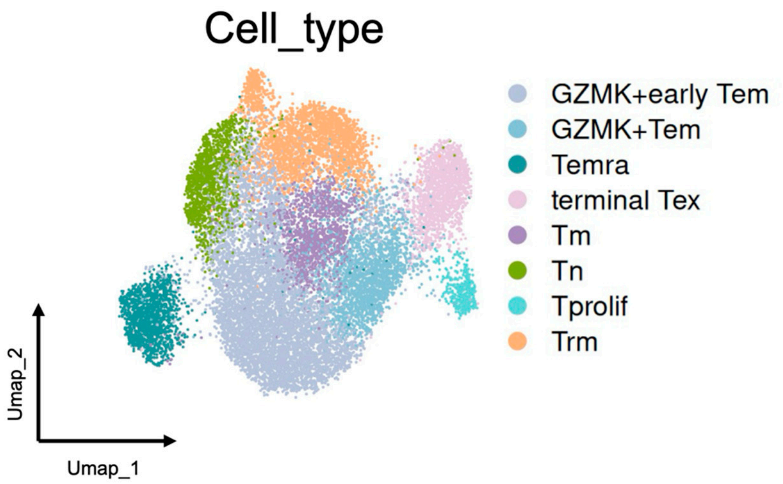

Figure S9. Transcriptional distribution of T-cell subpopulations in the PDAC dataset. UMAP projection of PDAC-infiltrating T cells included in the downstream trajectory analysis.

Table S2. Performance of the transfer-learned DNN in the held-out TIPC309, TIPC413, and TIPC416 samples, with patient-level bootstrap 95% confidence intervals.

group	condition	roc_auc	roc_auc_ ci_low	roc_auc_ ci_high	pr_auc	pr_auc_c i_low	pr_auc_c i_high	sensitivity	specificity	f1	brier
G1	w/o HLA	0.5404	0.5221	0.5779	0.5702	0.3470	0.5822	0.9391	0.2956	0.7338	0.3044
G1	+ HLA	0.7812	0.6862	0.7964	0.7387	0.5384	0.7781	0.8571	0.6200	0.7821	0.2032
G2	w/o HLA	0.6257	0.4969	0.6452	0.6396	0.3427	0.6719	0.2883	0.9645	0.4369	0.2795
G2	+ HLA	0.7318	0.6558	0.7445	0.7178	0.5379	0.7565	0.7202	0.7054	0.7276	0.2543
G3	w/o HLA	0.8379	0.5489	0.8605	0.7987	0.5414	0.8193	0.8022	0.7697	0.8002	0.1724
G3	+ HLA	0.8777	0.6608	0.8946	0.8693	0.5852	0.8796	0.8385	0.7908	0.8291	0.1747

Table S3. Patient-level paired bootstrap tests for metric differences between + HLA and w/o HLA models(*** indicates P < 0.001, ns indicates P > 0.05).

group	metric	value_wo	value_hla	delta	delta_ci_low	delta_ci_high	p_value	significance
G1	ROC-AUC	0.5404	0.7812	0.2408	0.1082	0.2577	0.000	***
G1	PR-AUC	0.5702	0.7387	0.1684	0.1564	0.1960	0.000	***
G1	Sensitivity	0.9391	0.8571	-0.0820	-0.1627	-0.0553	0.000	***
G1	Specificity	0.2956	0.6200	0.3244	0.1974	0.3421	0.000	***
G1	F1	0.7338	0.7821	0.0483	-0.0137	0.0610	0.222	ns
G2	ROC-AUC	0.6257	0.7318	0.1061	0.0903	0.2043	0.000	***
G2	PR-AUC	0.6396	0.7178	0.0782	0.0486	0.2254	0.000	***
G2	Sensitivity	0.2883	0.7202	0.4320	0.2651	0.4672	0.000	***
G2	Specificity	0.9645	0.7054	-0.2591	-0.3169	0.0482	0.222	ns
G2	F1	0.4369	0.7276	0.2907	0.1570	0.3998	0.000	***
G3	ROC-AUC	0.8379	0.8777	0.0398	0.0322	0.1542	0.000	***
G3	PR-AUC	0.7987	0.8693	0.0707	0.0438	0.2117	0.000	***
G3	Sensitivity	0.8022	0.8385	0.0363	-0.0214	0.2500	0.816	ns
G3	Specificity	0.7697	0.7908	0.0211	-0.1579	0.1974	0.302	ns
G3	F1	0.8002	0.8291	0.0289	0.0113	0.0979	0.000	***

Table S4. Software packages and computational environments used in this study.

Software/package	Version
**Monocle 2**	2.38.0
**Monocle 3**	1.4.26
**R**	4.5.1
**CellChat**	1.6.1
**Scanpy**	1.9.3
**Scirpy**	0.10.1
**scAtlasVAE**	1.0.5.9
**TCR-DeepInsight**	1.0.2.1
**PyTorch**	1.13.1
**CUDA**	11.7
**Python**	3.9.23
